# Supplementary material for: Exploitation of the fibrinolytic system by B-cell acute lymphoblastic leukemia and its therapeutic targeting
Source: Nat Commun. 2024 Nov 20;15:10059. doi: 10.1038/s41467-024-54361-4 (PMC11579293; doi:10.1038/s41467-024-54361-4)
Supplement: Supplementary file 1 — Supplementary Information [file 41467_2024_54361_MOESM1_ESM.pdf]

Supplemental Figure 1

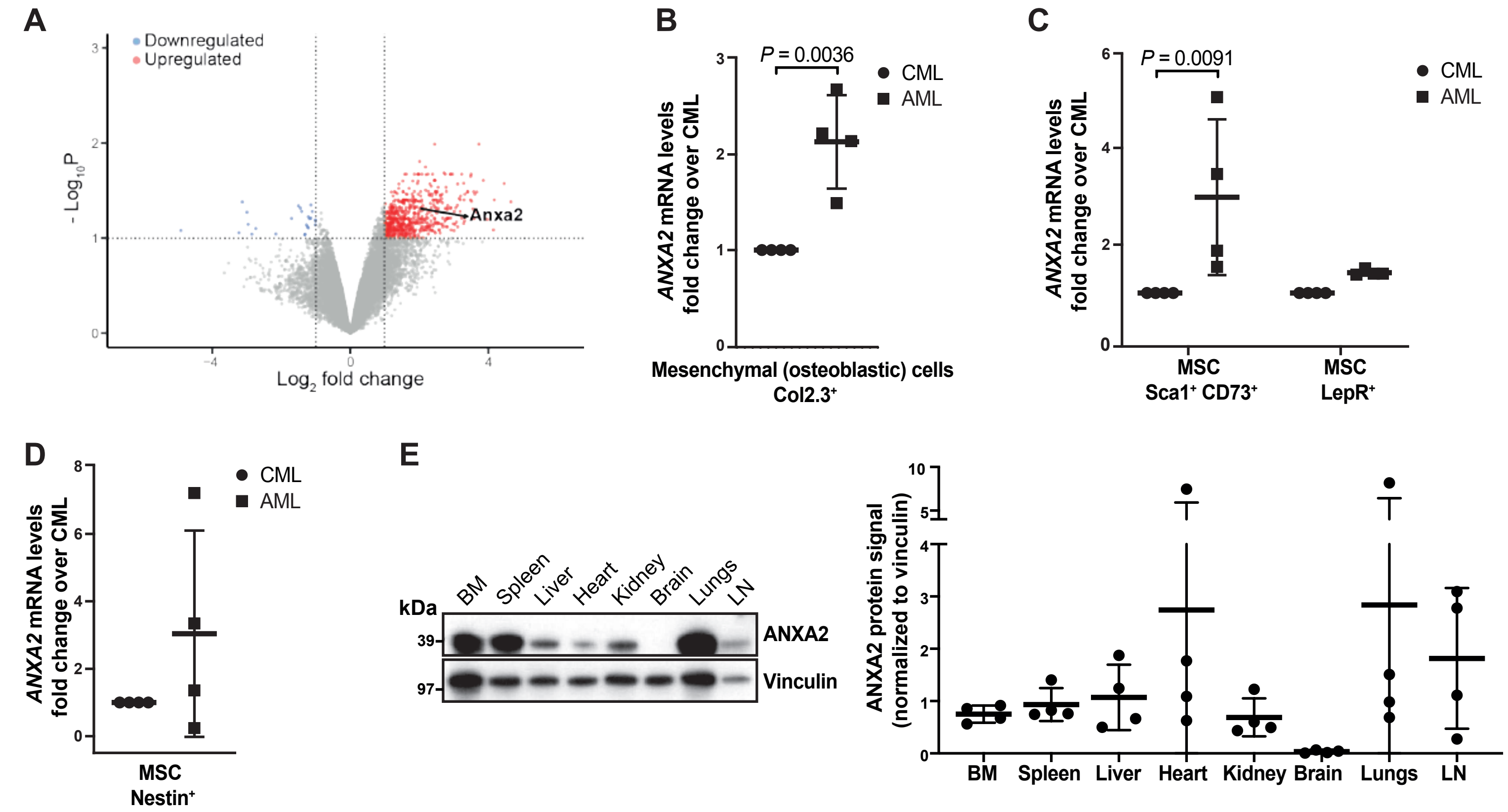

Supplemental Figure 1. (A) Volcano plot summarizing all differentially expressed genes in Col2.3kb-GFP⁺ mesenchymal (osteoblastic) cells from the bone marrow of mice with MLL-AF9⁺ acute myeloid leukaemia (AML) versus chronic myeloid leukemia (CML). Significantly upregulated and downregulated genes are shown in red versus blue, respectively. Annexin A2 is labeled. These data are shared with the manuscript Minciacchi et al., Blood Adv., 2024. (B-D) Relative expression levels of *Anxa2* by qRT-PCR in Col2.3kb-GFP⁺ mesenchymal (osteoblastic) cells (B), Sca1⁺ CD73⁺ and LepR⁺ mesenchymal stromal cells (MSC) (C) and Nestin⁺ MSC (D) from the bone marrow of mice with MLL-AF9⁺ AML versus CML (P = 0.0036, two-tailed t-test, P = 0.0091, two-way ANOVA, Sidak test, n=4, mean ± SD). (E) Representative immunoblot performed on lysates from the indicated tissues and probed with an antibody to annexin A2 (ANXA2) (38 kDa) (left). The quantification of the band intensity normalized to vinculin is shown on the right (n=4). BM = bone marrow; LN = lymph nodes. Source data are provided as a Source Data file.

Supplemental Figure 2

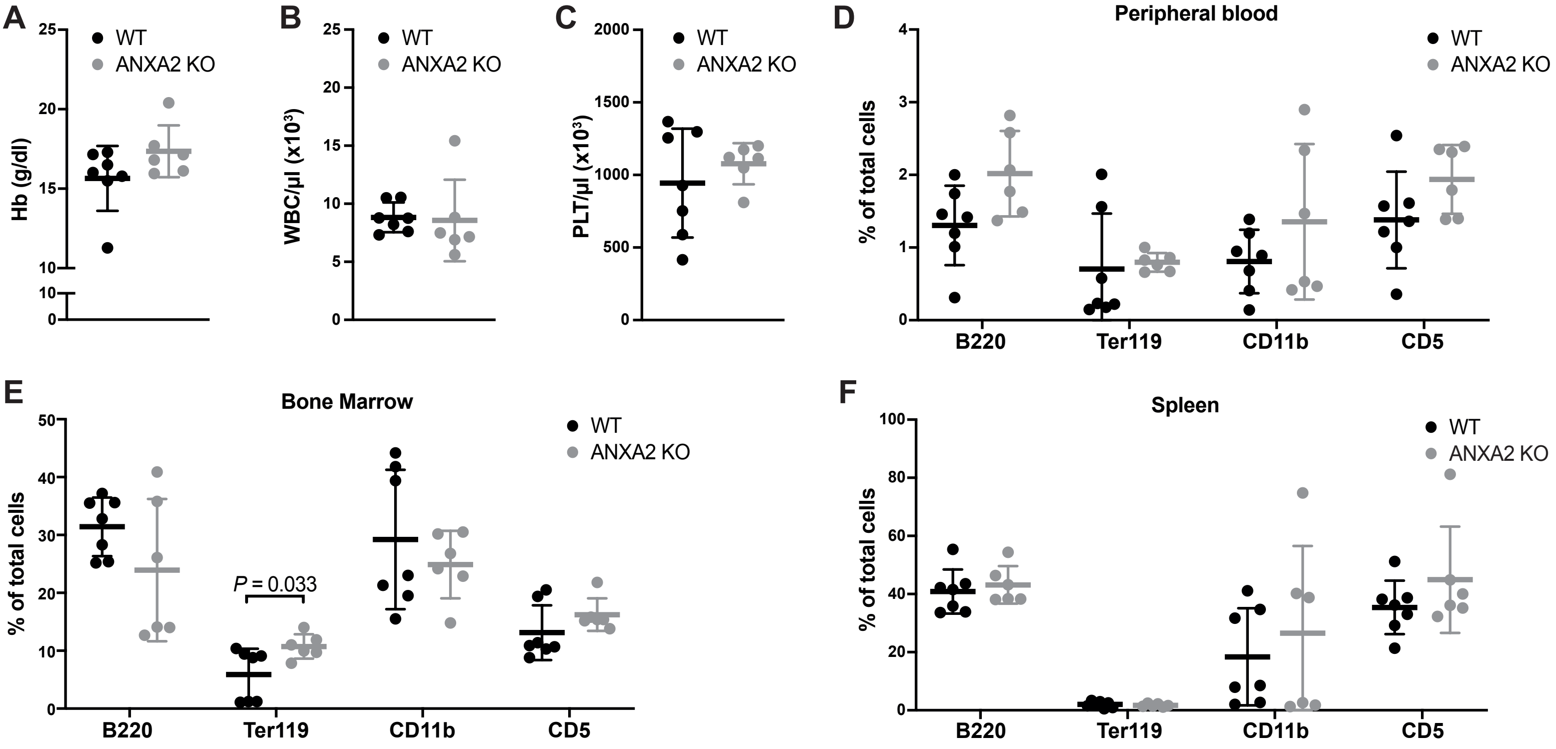

Supplemental Figure 2. (A-C) Hemoglobin (Hb) concentration (g/dl) (A), white blood cell (WBC) count per µl (B) and platelet (PLT) count per µl (C) in peripheral blood of normal WT (black) or ANXA2 KO (gray) mice as measured by complete blood count (CBC) analyzer (n=6, mean ± SD). (D-F) Percentage of cells positive for B220 (B cells), Terr119 (erythrocytes), CD11b (myeloid cells) or CD5 (T cells) of all cells in the peripheral blood (D), bone marrow (E) or spleen (F) of normal WT (black) or ANXA2 KO (gray) mice (multiple *t*-tests, n = 6 per group, mean ± SD). Source data are provided as a Source Data file.

Supplemental Figure 3

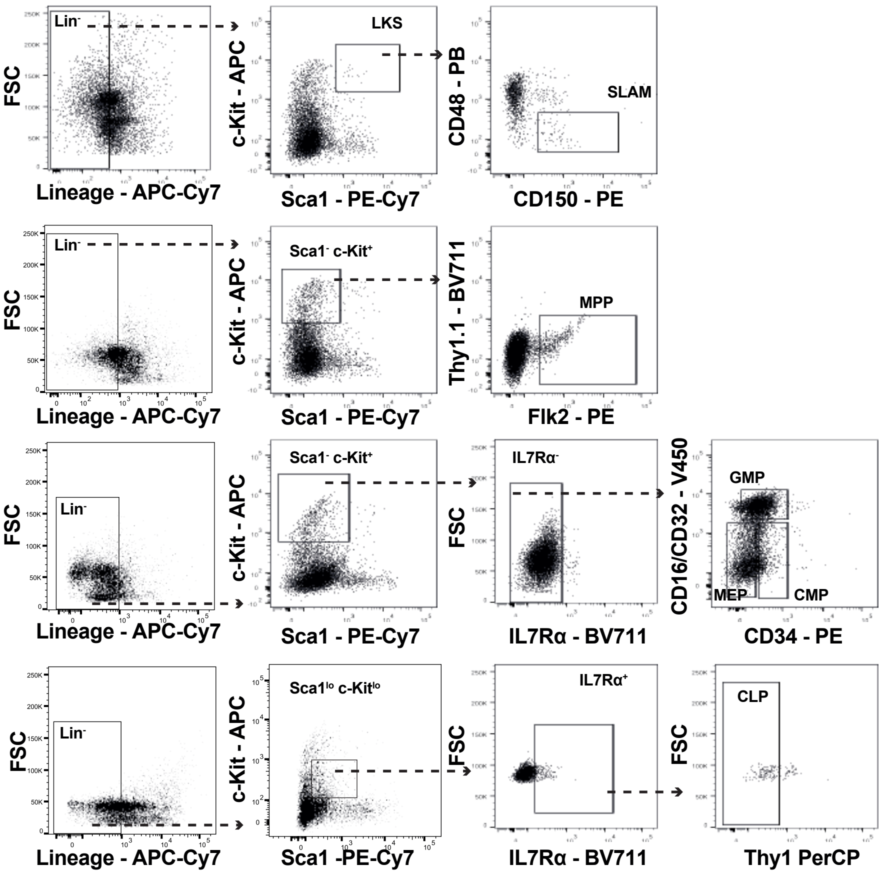

Supplemental Figure 3. Gating strategy for the analysis of Lin^{-} c-Kit^{+} Sca-1^{+} (LKS) and LKS CD48^{-} CD150^{+} (LKS SLAM) cells, Lin^{-} Thy1.1^{-} Sca-1^{+} c-Kit^{+} Flk2^{+} multipotent progenitors (MPP), IL7Rα^{-} Lin^{-} Sca-1^{-} c-Kit^{+} FcγR^{hi} CD34^{+} granulocyte-monocyte progenitors (GMP), IL7Rα^{-} Lin^{-} Sca-1^{-} c-Kit^{+} FcγR^{lo} CD34^{+} common myeloid progenitors (CMP), and IL7Rα^{-} Lin^{-} Sca-1^{-} c-Kit^{+} FcγR^{lo} CD34^{-} megakaryocyte-erythroid progenitors (MEP) and Lin^{-} IL7Rα^{+} Thy1.1^{-} Sca-1^{lo} c-Kit^{lo} common lymphoid progenitors (CLP).

Supplemental Figure 4

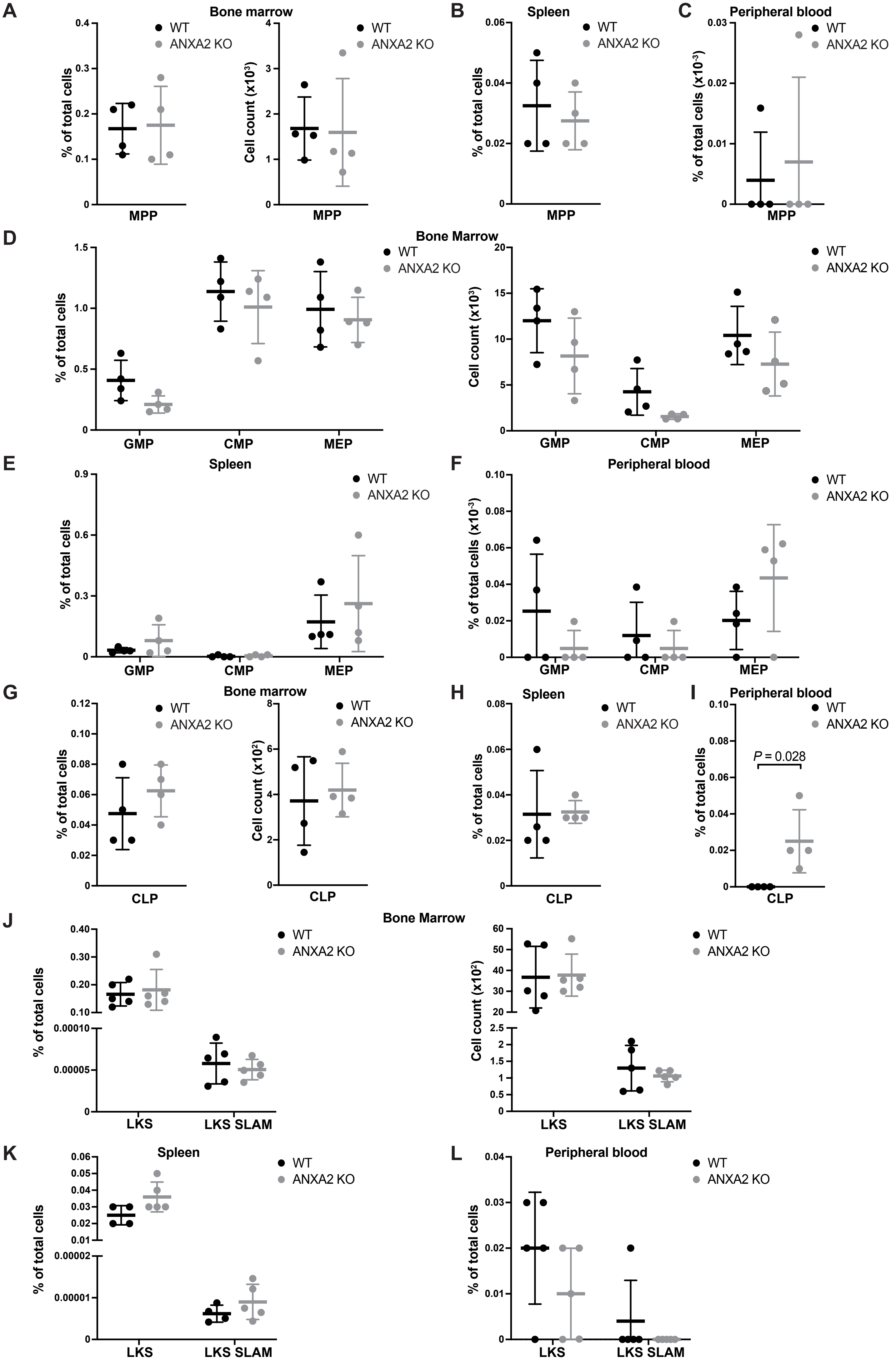

Supplemental Figure 4. (A) Percentage (left) and total cell count (right) of Lin$^-$ c-Kit^{hi} Sca-1^{hi} Thy1.1$^-$ Flk2$^+$ MPP cells in the bone marrow (half a femur) of normal WT (black) or ANXA2 KO (gray) mice (n=4, mean ± SD). (B-C) Percentage of Lin$^-$ c-Kit^{hi} Sca-1^{hi} Thy1.1$^-$ Flk2$^+$ MPP cells in the spleen (B) or peripheral blood (C) of normal WT (black) or ANXA2 KO (gray) mice (n=4, mean ± SD). (D) Percentage (left) and total cell count (right) of Lin$^-$ Sca-1$^-$ c-Kit$^+$ IL7Ra$^-$ FcyR^{hi} CD34$^+$ granulocyte-monocyte progenitors (GMP), Lin$^-$ Sca-1$^-$ c-Kit$^+$ IL7Rα$^-$ FcyR^{lo} CD34$^+$ common myeloid progenitors (CMP) and Lin$^-$ Sca-1$^-$ c-Kit$^+$ IL7Rα$^-$ FcyR^{lo} CD34$^-$ megakaryocyte-erythrocyte progenitor (MEP) cells of all cells in the bone marrow of normal WT (black) or ANXA2 KO (gray) mice. (E-F) Percentage of Lin$^-$ Sca-1$^-$ c-Kit$^+$ IL7Ra$^-$ FcyR^{hi} CD34$^+$ granulocyte-monocyte progenitors (GMP), Lin$^-$ Sca-1$^-$ c-Kit$^+$ IL7Rα$^-$ FcyR^{lo} CD34$^+$ common myeloid progenitors (CMP) and Lin$^-$ Sca-1$^-$ c-Kit$^+$ IL7Rα$^-$ FcyR^{lo} CD34$^-$ megakaryocyte-erythrocyte progenitor (MEP) cells of all cells in the spleen (E) or peripheral blood (F) of normal WT (black) or ANXA2 KO (gray) mice (n=4, mean ± SD). (G) Percentage (left) and total cell count (right) of Lin$^-$ Sca-1^{lo} c-Kit^{lo} IL7Rα$^+$ Thy1.1$^-$ common lymphoid progenitor (CLP) cells of all cells in the bone marrow of normal WT (black) or ANXA2 KO (gray) mice (n=4). (H-I) Percentage of Lin$^-$ Sca-1^{lo} c-Kit^{lo} IL7Rα$^+$ Thy1.1$^-$ common lymphoid progenitor (CLP) cells of all cells in the spleen (H) or peripheral blood (I) of normal WT (black) or ANXA2 KO (gray) mice (two-tailed *t*-test, n=4, mean ± SD). (J) Percentage (left) and total cell count (right) of Lin$^-$ c-Kit$^+$ Sca-1$^+$ (LKS) and LKS CD48$^-$ CD150$^+$ (LKS SLAM) cells in the bone marrow of normal WT (black) or ANXA2 KO (gray) mice (n=6, mean ± SD). (K-L) Percentage of Lin$^-$ c-Kit$^+$ Sca-1$^+$ (LKS) and LKS CD48$^-$ CD150$^+$ (LKS SLAM) cells in the spleen (K) or peripheral blood (L) of normal WT (black) or ANXA2 KO (gray) mice (n=6, mean ± SD). Source data are provided as a Source Data file.

Supplemental Figure 5

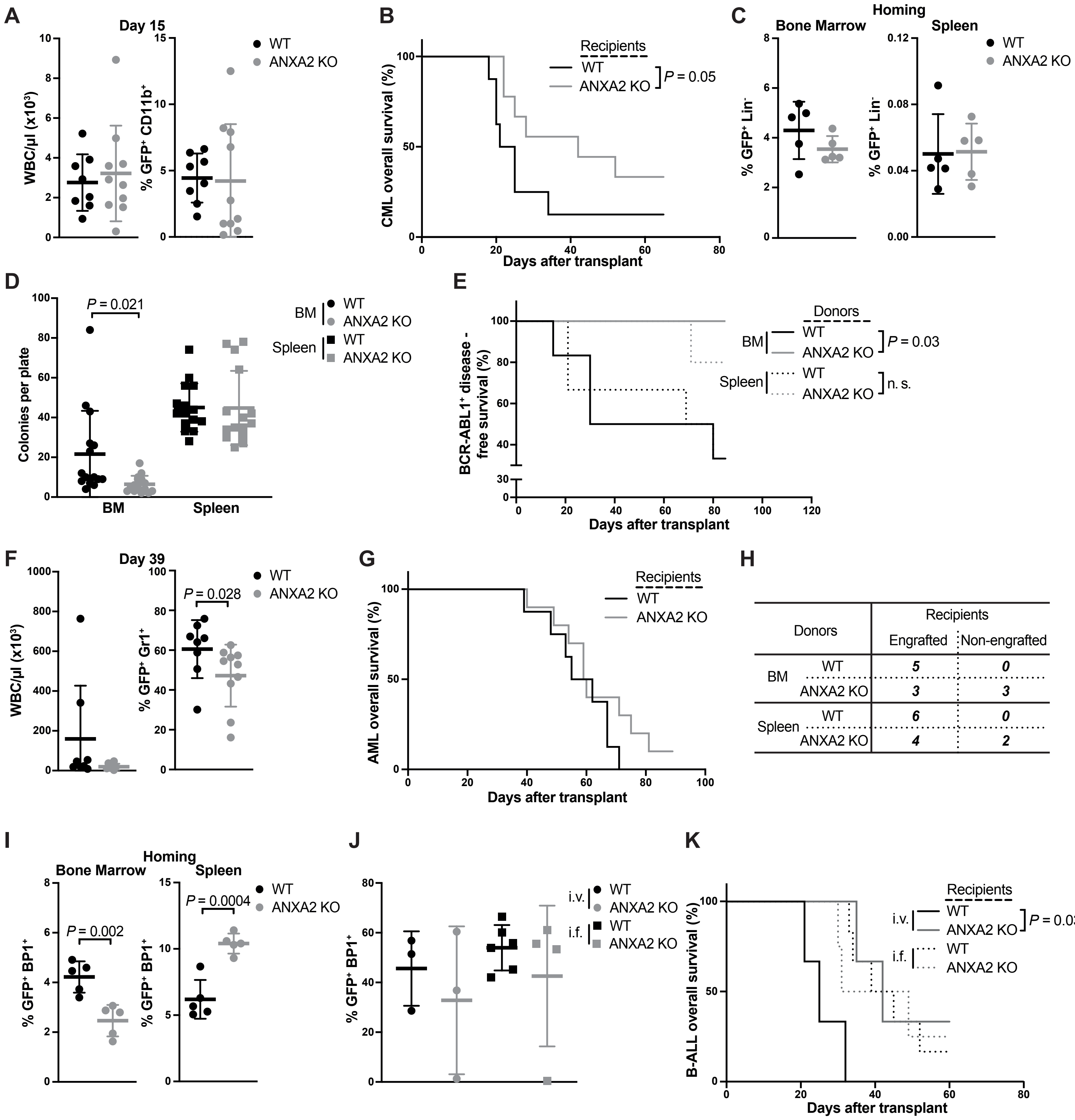

Supplemental Figure 5. (A) White blood cell count (WBC) per µl (left) and percentage of GFP (BCR-ABL1)$^+$ CD11b$^+$ myeloid cells (right) of all cells in the peripheral blood (PB) of WT (black) or ANXA2 KO (gray) mice transplanted with CML-initiating cells, collected on day 15 after transplantation (WT n=8, ANXA2 KO n=9, mean ± SD). (B) Kaplan-Meier-style survival curve of wildtype (WT; black) or ANXA2-deficient (ANXA2 KO; gray) recipient mice with CML (P = 0.05, Wilcoxon test, WT n=8, ANXA2 KO n=9). (C) Percentage of GFP (BCR-ABL1)$^+$ Lin$^-$ CML-initiating cells of all cells which had homed to the bone marrow (left) or spleen (right) of WT (black) or ANXA2 KO (gray) mice within 18 hours after transplantation (n=5, mean ± SD). (D) Number of colonies per plate derived from total bone marrow (circles) or spleen (squares) cells from WT (black) or ANXA2 KO (gray) recipient mice with CML (P = 0.021, two-way ANOVA, Sidak test, n=15, mean ± SD). $2x10^4$ total bone marrow and $2x10^5$ total spleen cells had been plated. Colonies were scored on day 7 after plating. (E) Kaplan-Meier-style survival curve of WT secondary recipient mice of $3x10^6$ unsorted total BM (solid line) or $3x10^6$ spleen (dotted line) cells from WT (black) or ANXA2 KO (gray) donor mice with established CML (P = 0.03, Log-rank test, WT n=6, ANXA2 KO n=5, mean ± SD) succumbing to BCR-ABL1$^+$ disease (CML, B-ALL

or mixtures of both, as frequently described in secondary recipients of CML cells in this murine model). (F) WBC count per µl (left) and percentage of GFP (MLL-AF9)$^+$ Gr1$^+$ cells of all cells (right) in the peripheral blood of WT (black) or ANXA2 KO (gray) recipient mice with MLL-AF9$^+$ AML, collected on day 39 after transplantation (P = 0.028, two-tailed Mann Whitney test, n=8-10). (G) Kaplan-Meier-style survival curve of WT (black) or ANXA2 KO (gray) recipient mice with MLL-AF9$^+$ AML (WT n=8, ANXA2 KO n=10, mean ± SD). (H) Number of WT secondary recipient mice which engrafted or died of engraftment failure after transplantation of 3x10^6 bone marrow or spleen cells from primary WT or ANXA2 KO mice with BCR-ABL1$^+$ B-ALL (n=5-6). (I) Percentage of GFP (BCR-ABL1)$^+$ BP1$^+$ B-ALL-initiating cells which had homed to the bone marrow (left) or spleen (right) of WT (black) or ANXA2 KO (gray) mice within 18 hours after transplantation (P = 0.002, P = 0.0004, two-tailed *t*-test, n=5, mean ± SD). (J) Percentage of GFP (BCR-ABL1)$^+$ BP1$^+$ cells of all cells in the PB of WT (black) or ANXA2 KO (gray) recipient mice with BCR-ABL1$^+$ B-ALL on day 20 after transplantation. 1x10^6 bone marrow cells had been injected either intravenously (circles) or intrafemorally (squares) (WT i.v. n=3, ANXA2 KO i.v. n=3, WT i.f. n=6 and ANXA2 KO i.f. n=4, mean ± SD). (K) Kaplan-Meier-style survival curve of WT (black) or ANXA2 KO (gray) recipient mice with BCR-ABL1$^+$ B-ALL. BM cells had been injected either intravenously (solid line) or intrafemorally (dotted line) as in G (P = 0.03, Log-rank test, i.v. n=3, ANXA2 KO i.v. n=3, WT i.f. n=6 and ANXA2 KO i.f. n=4). Source data are provided as a Source Data file.

Supplemental Figure 6

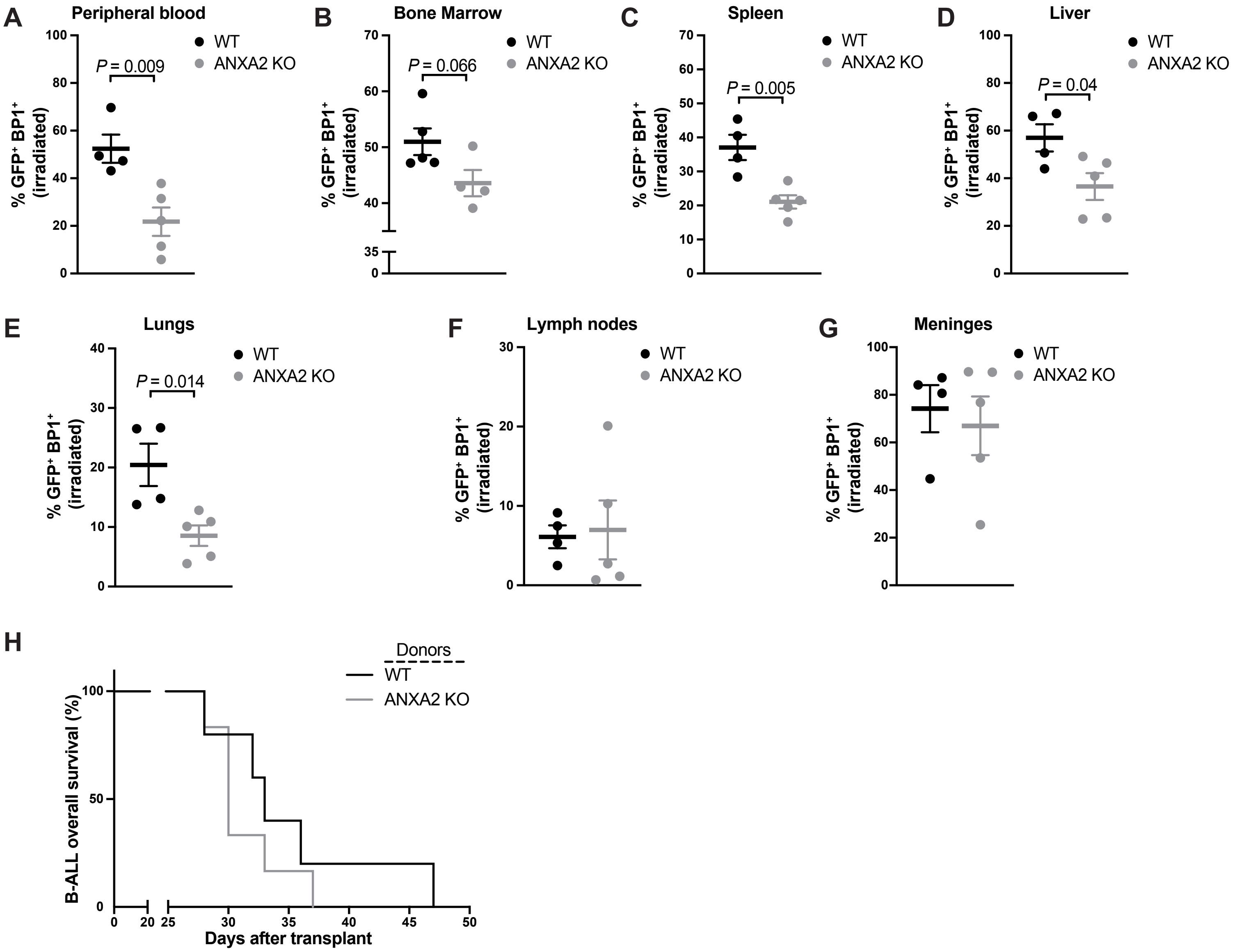

Supplemental Figure 6. (A-G) Percentage of GFP (BCR-ABL1)$^+$ BP1$^+$ cells of all cells in the peripheral blood (A), bone marrow (B), spleen (C), liver (D), lungs (E), lymph nodes (F) and meninges (G) of WT (black) and ANXA2 KO (gray) irradiated (2x450 cGy) recipient mice with BCR-ABL1$^+$ B-ALL on day 12 after transplantation. 1x10^6 bone marrow cells transduced with BCR-ABL1 had been transplanted (P = 0.009, P = 0.066, P = 0.005, P = 0.04, P = 0.014, two-tailed *t*-test, n=4, mean ± SD). (H) Kaplan-Meier-style survival curve of WT recipients transplanted with 1x10^6 BCR-ABL1-transduced bone marrow cells from WT (black) or ANXA2 KO (gray) mice (n=5) in the B-ALL model. Source data are provided as a Source Data file.

Supplemental Figure 7

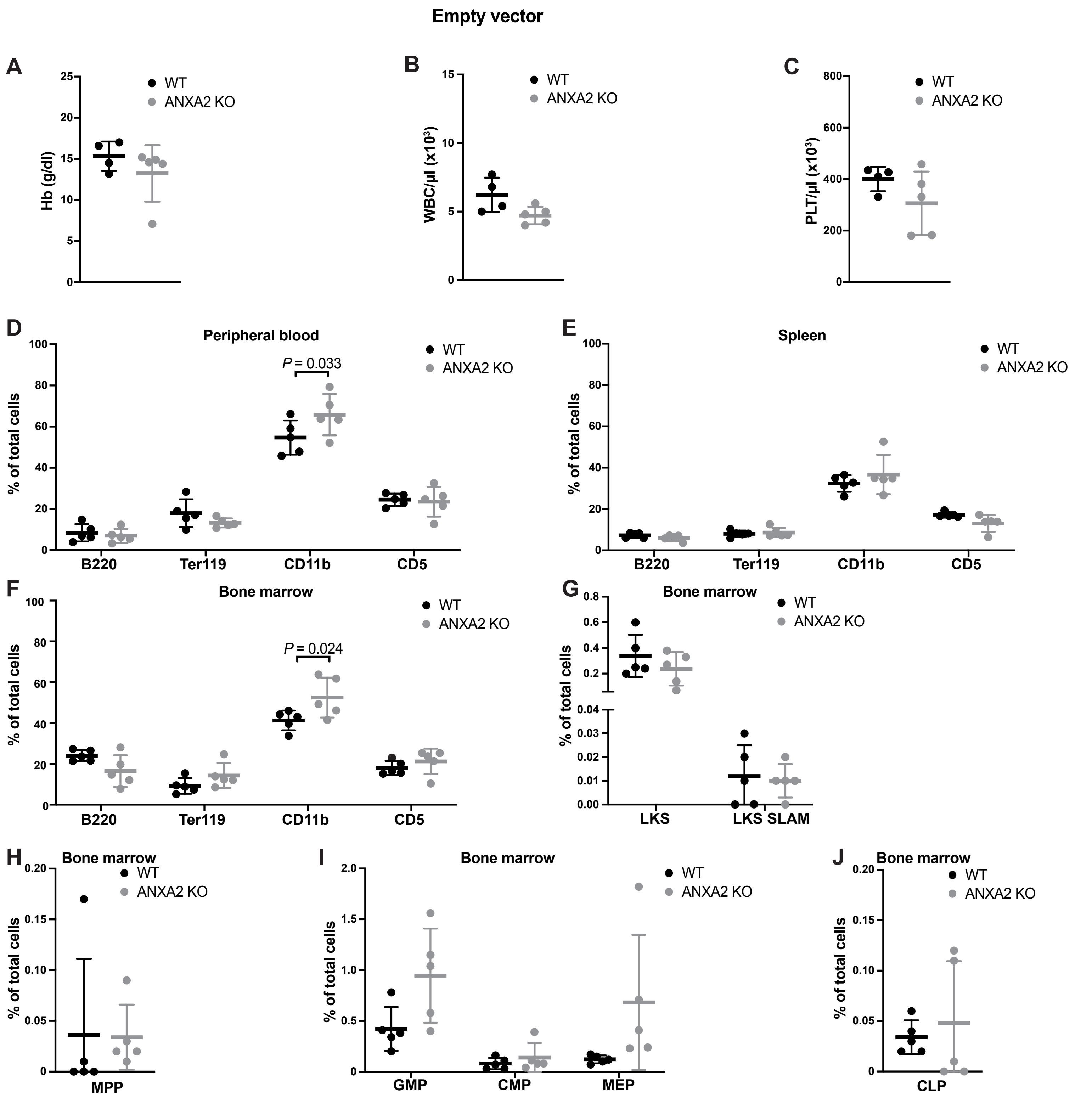

Supplemental Figure 7. (A-C) Hemoglobin (Hb) concentration (A), white blood cell count (WBC) count per µl (B) and platelet (PLT) count per µl (C) in peripheral blood of WT (black) or ANXA2 KO (gray) mice transplanted with $2.5x10^5$ 5-fluorouracil-pretreated bone marrow cells transduced with empty vector (MSCV-IRES GFP)-expressing retrovirus (WT n=4, ANXA2 KO n=5, mean ± SD). (D-F) Percentage of cells positive for B220 (B cells), Terr119 (erythrocytes), CD11b (myeloid cells) and CD5 (T cells) of all cells in the peripheral blood (D), spleen (E) and bone marrow (F) of WT (black) or ANXA2 KO (gray) mice transplanted with $2.5x10^5$ bone marrow cells transduced with empty vector (MSCV-IRES GFP)-expressing retrovirus (*P* = 0.033, two-way ANOVA, Sidak test, n=5, mean ± SD). (G-J) Percentage of LKS and LKS SLAM (G), MPP (H), GMP, CMP and MEP (I) and CLP (J) of all cells in the bone marrow of WT (black) or ANXA2 KO (gray) mice transplanted with 2.5×10^5 bone marrow cells transduced with empty vector (MSCV-IRES GFP)-expressing retrovirus (n=5, mean ± SD). Source data are provided as a Source Data file.

Supplemental Figure 8

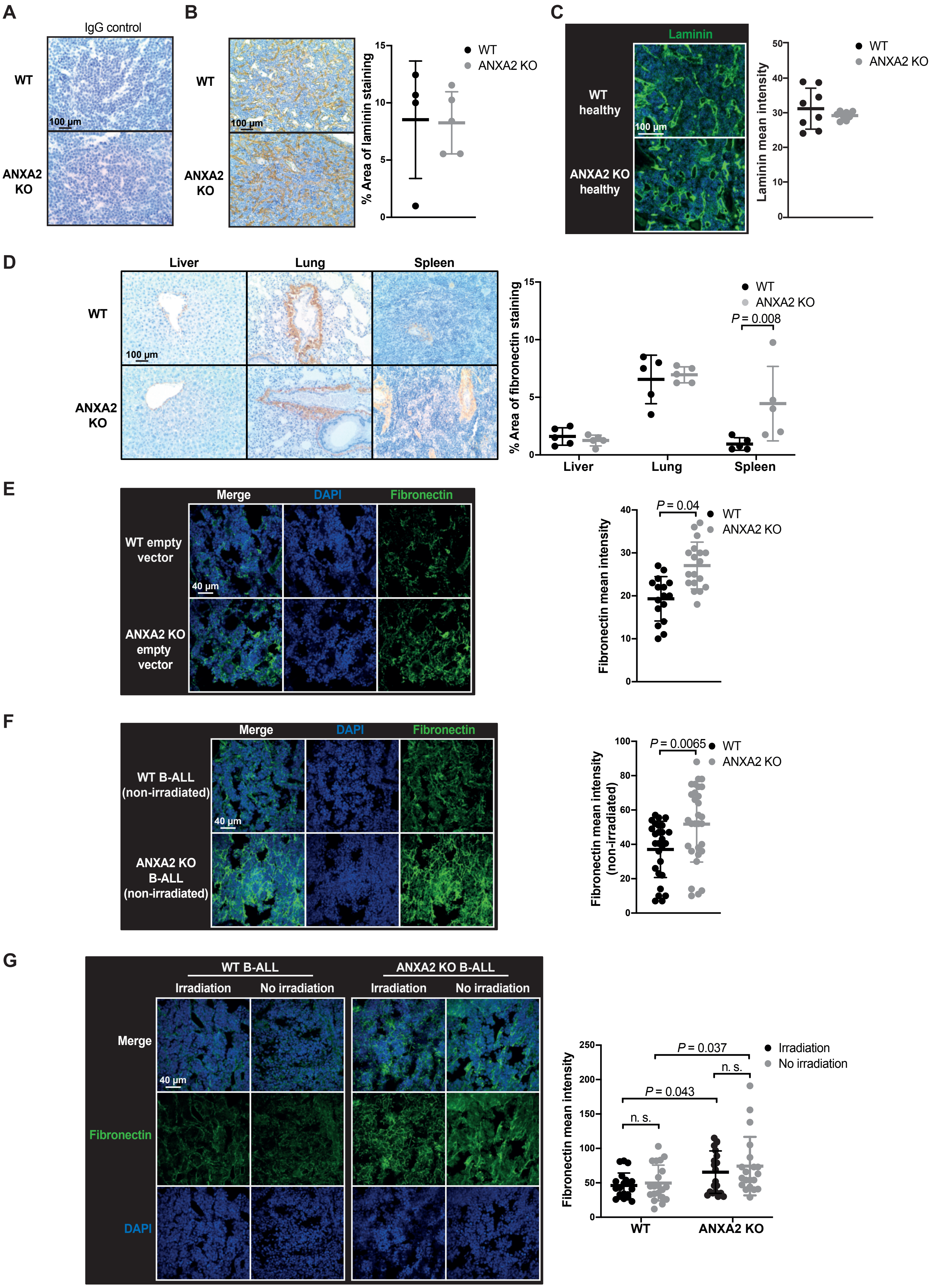

Supplemental Figure 8. (A) Immunohistochemistry (IHC) for IgG control (detected by immunoperoxidase using yellow-brown horseradish-peroxidase chromogen) in bone sections from WT or ANXA2 KO recipient mice with BCR-ABL1$^+$ B-ALL. The scale bar depicts 100 µm. (B) Immunohistochemistry (IHC) (left) for laminin (detected by immunoperoxidase using yellow-brown horseradish-peroxidase chromogen) and its quantification (right) in bone sections of WT (black) or ANXA2 KO (gray) recipient mice with BCR-ABL1$^+$ B-ALL. The scale bar represents 100 µm. (WT n=4, ANXA2 KO n=5, mean ± SD). (C) IF images of bone sections from healthy WT or ANXA2 KO mice, stained for laminin (n=4, mean ± SD). The scale bar depicts 40 µm. (D) IHC (left) for fibronectin (detected by immunoperoxidase using yellow-brown horseradish-peroxidase chromogen) and its quantification (right) in liver, lung and spleen from WT (black) or ANXA2 KO (gray) recipient mice with BCR-ABL1$^+$ B-ALL (P = 0.008, two-way ANOVA, Sidak test, n=5, mean ± SD). The scale bar depicts 100 µm. (E) Immunofluorescence (IF) (left) for fibronectin (green) and 4′,6-diamidino-2-phenylindole (DAPI) (blue) and its quantification (right) in bone sections of WT (black) or ANXA2 KO (gray) recipient mice transplanted with $2.5x10^5$ bone marrow cells transduced with empty vector (MSCV-IRES GFP)-expressing retrovirus (P = 0.04,two-tailed *t*-test, WT n=4, ANXA2 KO, n=5, mean ± SD). The scale bar depicts 40 µm. (F) IF (left) for fibronectin (green) and DAPI (blue) and its quantification (right) in bone sections of non-irradiated WT (black) or ANXA2 KO (gray) recipient mice with BCR-ABL1$^+$ B-ALL (P = 0.0065, two-tailed Mann Whitney test, WT n=8, ANXA2 KO n=6, mean ± SD). The scale bar depicts 40 µm. (G) IF (left) for fibronectin (green) and DAPI (blue) and its quantification (right) in bone sections from non-irradiated and irradiated (2x450 cGy) WT (black) or ANXA2 KO (gray) recipient mice with BCR-ABL1$^+$ B-ALL on day 24 and 20, respectively (P = 0.037, P = 0.043, two-way ANOVA, Sidak test, n=5, mean ± SD). Source data are provided as a Source Data file.

Supplemental Figure 9

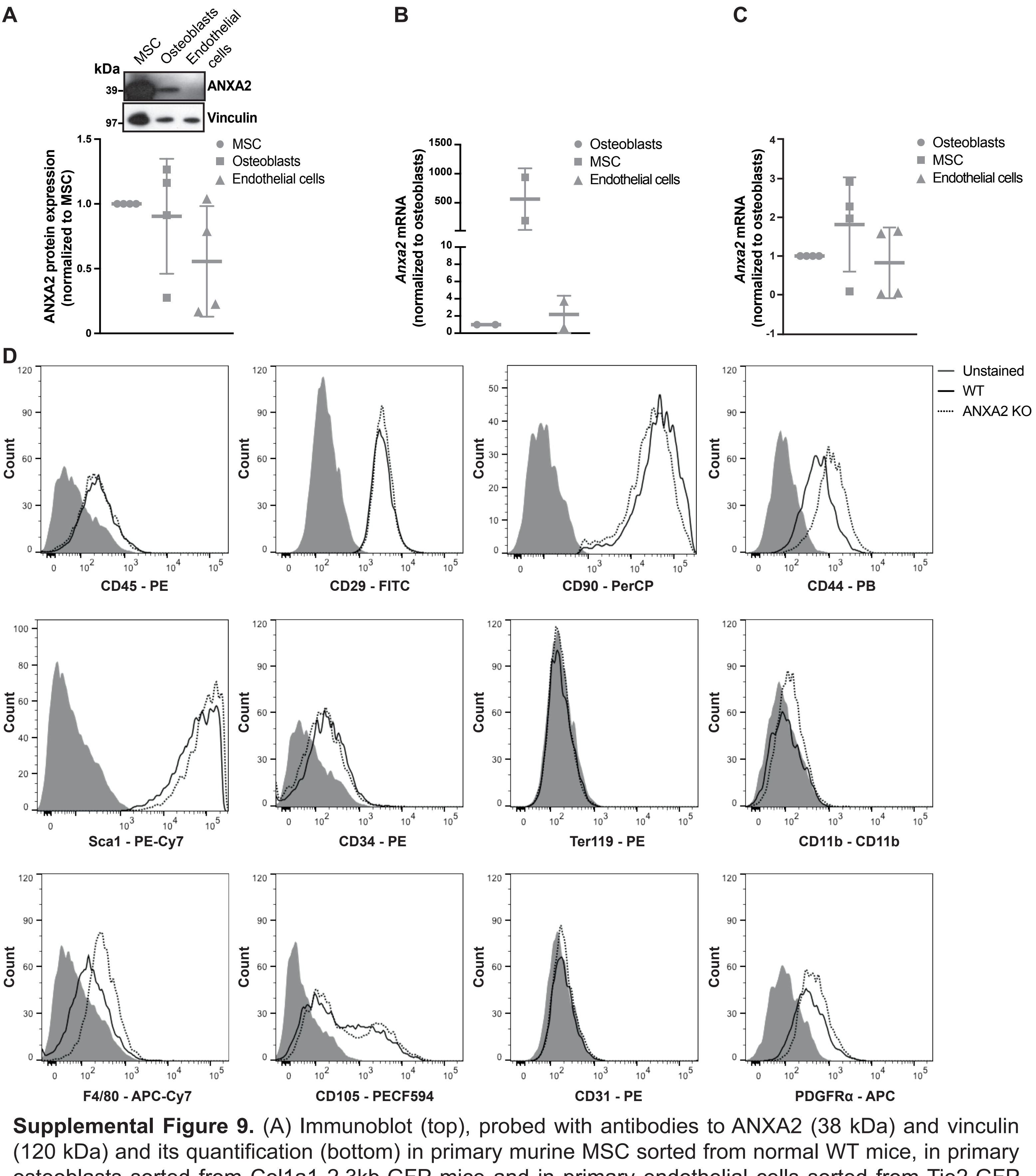

Supplemental Figure 9. (A) Immunoblot (top), probed with antibodies to ANXA2 (38 kDa) and vinculin (120 kDa) and its quantification (bottom) in primary murine MSC sorted from normal WT mice, in primary osteoblasts sorted from Col1a1 2.3kb-GFP mice and in primary endothelial cells sorted from Tie2-GFP mice (n=4, mean ± SD). (B) RT-qPCR for *Anxa2* in primary murine osteoblasts sorted from Col1a1 2.3kb-GFP mice, in primary murine MSC sorted from Nestin-GFP mice and in primary endothelial cells sorted from Tie2-GFP mice (n=2, mean ± SD). These data were normalized to *Gapdh* as housekeeping control. (C) RT-qPCR for *Anxa2* in primary murine osteoblasts (CD4$^-$ CD8$^-$ Ter119$^-$ CD11b$^-$ Gr1$^-$ CD31$^-$ Sca1$^-$ CD51$^+$), in primary murine MSC (CD45$^-$ Ter119$^-$ Sca1$^+$ PDGFRα$^+$) sorted from WT mice and in primary endothelial cells (CD31$^+$) (n=5, mean ± SD).These data were normalized to *Gapdh* as housekeeping control. (D) Expression of the indicated surface markers, analyzed by flow cytometry, on sorted primary murine MSC from normal WT (black line) and ANXA2 KO (gray dotted line) mice. The shaded area represents unstained cells. Source data are provided as a Source Data file.

Supplemental Figure 10

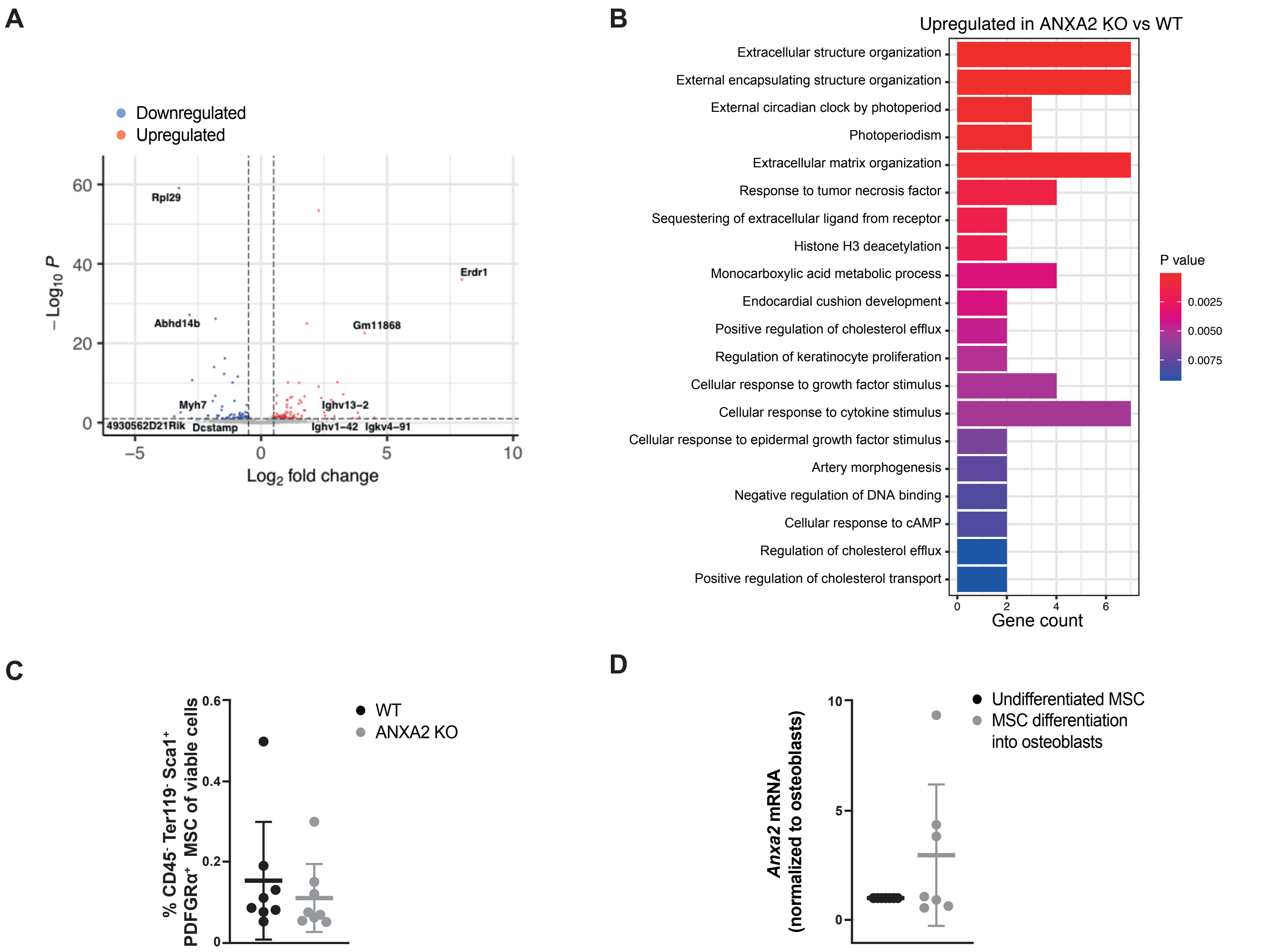

Supplemental Figure 10. (A) Volcano plot summarizing all differentially expressed genes in sorted mesenchymal stromal cells (MSC) from the bone marrow of normal WT or ANXA2 KO mice. Significantly upregulated and downregulated genes are shown in red and blue, respectively. (B) Gene ontology analysis of significantly upregulated genes in MSC from the bone marrow of healthy WT or ANXA2 KO mice. The bar graph summarizes non-redundant terms with P values provided by the color coding. (C) Percentage of $CD45^-$ $Ter119^-$ $Sca1^+$ $PDFGR\alpha^+$ MSC in the bone marrow of healthy WT or ANXA2 KO mice (n=8, mean ± SD). (D) RT-qPCR for *Anxa2* in primary murine MSC with or without *in vitro* differentiation into osteoblasts (n=7, mean ± SD). Source data are provided as a Source Data file.

Supplemental Figure 11

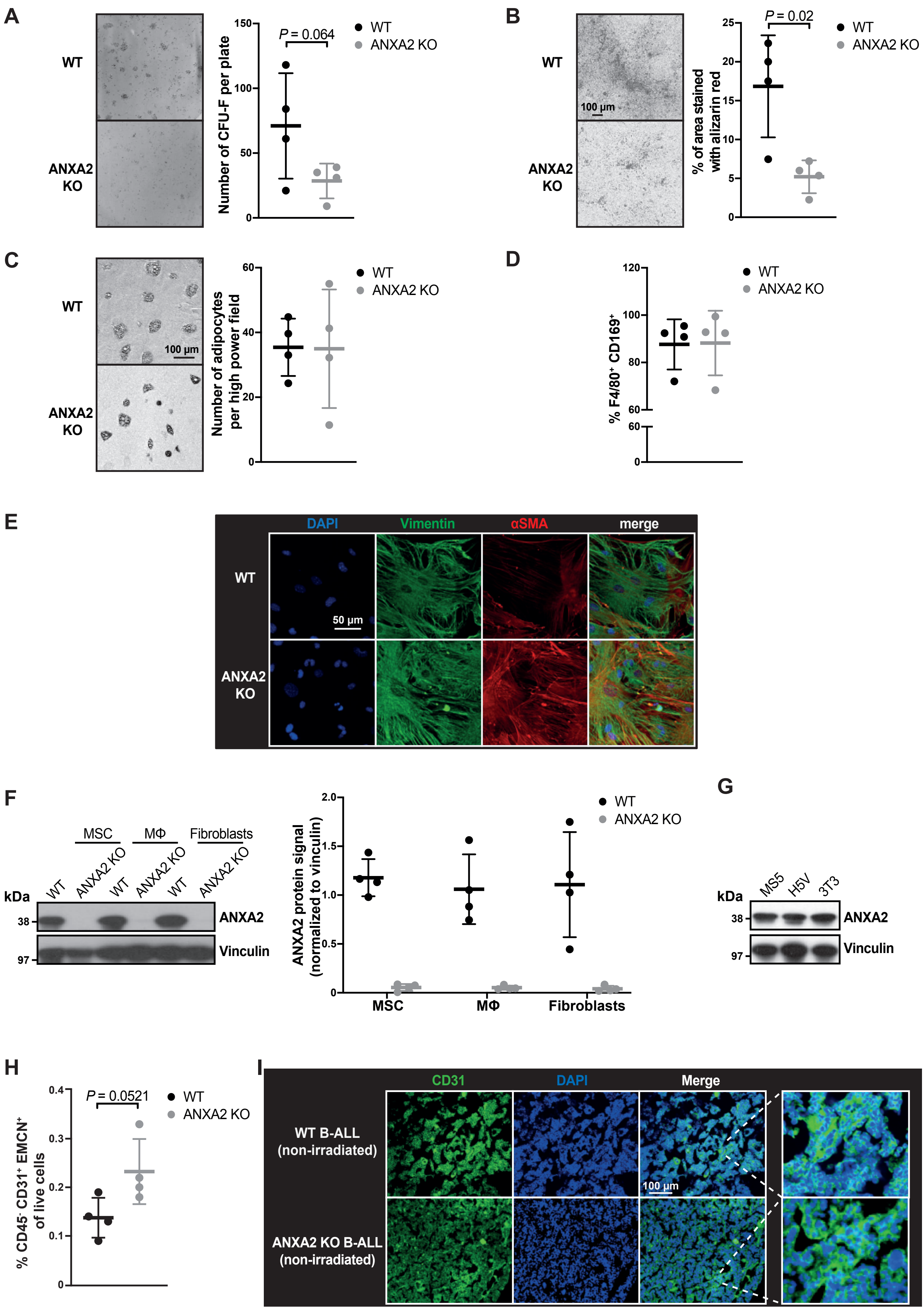

Supplemental Figure 11. (A) Representative images (left) and their quantification (right) of fibroblast colony-forming units (CFU-F) from cultured primary murine mesenchymal stromal cells (MSC) isolated from normal WT (black) or ANXA2 KO (gray) mice. $2x10^3$ cells had been plated in a 10 cm plate. Staining was performed using crystal violet (P = 0.064, two-tailed t-test, n=4, mean ± SD). (B) Representative images (left) and their quantification (right) of osteogenic differentiation of primary murine MSC from normal WT (black) or ANXA2 KO (gray) mice. Alizarin red staining was performed to detect calcium deposits (P = 0.02, two-tailed t-test, n=4, mean ± SD). $5x10^3$ cells had been plated. The scale bar depicts 100 µm. (C) Representative images (left) and their quantification (right) of adipogenic differentiation of primary MSC from normal WT (black) or ANXA2 KO (gray) mice. Oil red O staining was performed to detect lipid-rich droplets in adipocytes (n=4, mean ± SD). 5×10^3 cells had been plated. The scale bar depicts 100 µm. (D) Percentage of $F4/80^+$ $CD169^+$ cells of all cells in cultured primary murine macrophages (MΦ) isolated from the bones of normal WT (black) or ANXA2 KO (gray) mice (n=4, mean ± SD). (E) Representative immunofluorescence images of primary fibroblasts isolated from the bones of normal WT or ANXA2 KO mice stained for vimentin (green), α smooth muscle actin (SMA) (red) and DAPI (blue) (n=4). The scale bar depicts 50 µm. (F) Representative immunoblot of lysates from primary MSC, macrophages (MΦ) and fibroblasts from normal WT (black) or ANXA2 KO (gray) mice, probed with antibodies to ANXA2 (38 kDa) and vinculin (120 kDa). The quantification (WT (black) and ANXA2 KO (gray)) is shown on the right (n=4, mean ± SD). (G) Representative immunoblot of lysates of murine stroma (MS5), endothelial (H5V) and fibroblastic (3T3) cell lines, probed with antibodies to ANXA2 (38 kDa) and vinculin (120 kDa). H5V cells are endothelial cells, derived from embryonic heart. (H) Percentage of $CD45^-$ $CD31^+$ $EMCN^+$ endothelial cells in the bone marrow of healthy WT or ANXA2 KO mice (P = 0.0521, two-tailed t-test, n=4, mean ± SD). (I) IF images stained for CD31 (endothelial cells, green) and 4′,6-diamidino-2-phenylindole (DAPI, blue) in bone sections of non-irradiated WT or ANXA2 KO mice with B-ALL. The scale bar represents 100 µm. Source data are provided as a Source Data file.

Supplemental Figure 12

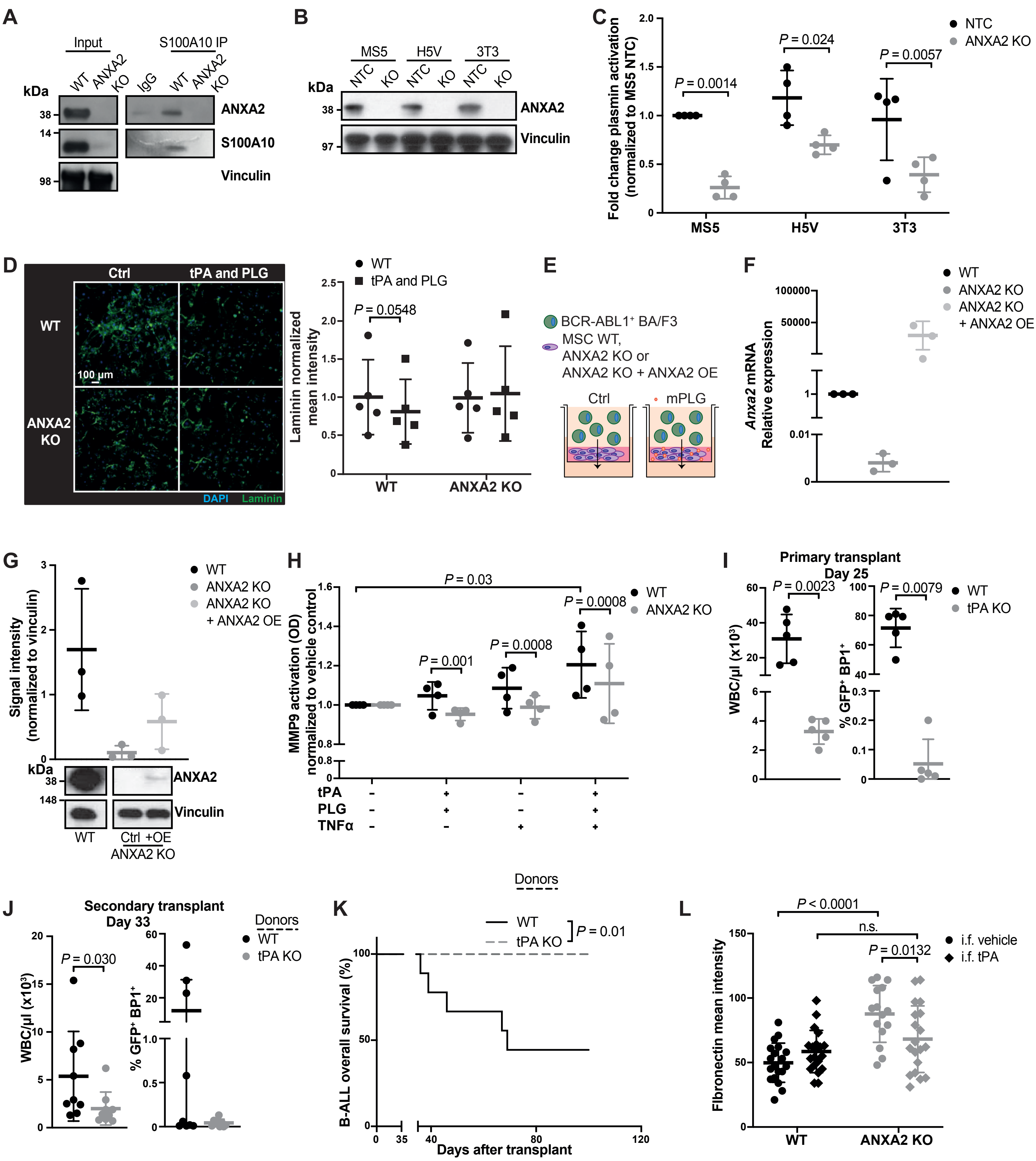

Supplemental Figure 12. (A) Immunoblot for ANXA2 and S100A10 using lysates of WT or ANXA2 KO MSC, in which an anti-S100A10 antibody was used to co-immunoprecipitate ANXA2. The input is shown on the left, and vinculin was used as loading control. IP = immunoprecipitation. (B) Representative immunoblot of lysates from murine stroma (MS5), endothelial (H5V) and fibroblastic (3T3) cell lines, probed with antibodies to ANXA2 (38 kDa) and vinculin (120 kDa), after knockout (KO) of *Anxa2* versus no template control (NTC). (C) Levels of active plasmin, measured as the ability to cleave a colorogenic substrate 5 minutes after addition of tissue plasminogen activator (tPA) and the plasmin precursor plasminogen (PLG) to the medium of MS5, H5V and 3T3 cell lines after knockout (KO) of *Anxa2* versus no template control (NTC). The data are normalized to the WT control. (P = 0.0014, P = 0.024, P = 0.0057, two-way ANOVA, Sidak test, n=4, mean ± SD). (D) Immunofluorescence images of laminin (green) and DAPI (blue) staining in cultures of WT or ANXA2 KO MSC with or without activation of plasmin by tissue plasminogen activator (tPA) and plasminogen (PLG) (n=5, mean ± SD). The green signal intensity is normalized by the number of cells identified by DAPI staining. The scale bar depicts 100 µm. (E) Schematic representation of the invasion experiment presented in Figure 2D. (F) RT-qPCR for *Anxa2* in WT (black) and ANXA2 KO MSC (dark gray) and ANXA2 KO MSC with transient overexpression (OE) of *Anxa2* (light gray) 48 hours after transfection (n=3, mean ± SD). (G) Immunoblot of lysates of WT (black) and ANXA2 KO MSC (dark gray) and ANXA2 KO MSC with transient overexpression (OE) of *Anxa2* (light gray) 48 hours after transfection (n=3, mean ± SD). The quantification is shown on the top. (H) Levels of active matrix metalloproteinase (MMP9), measured as the ability to cleave a colorogenic substrate after addition of tissue plasminogen activator (tPA) and the plasmin precursor plasminogen (PLG) to the medium of primary WT (black) or ANXA2 KO (gray) MSC with or without treatment with tumor necrosis factor α (TNFα) to stimulate MMP9-expression (P = 0.03, P = 0.001, P = 0.0008, P = 0.0008, two-way ANOVA, Sidak test, n=4, mean ± SD). The data are normalized to the control. OD = optical density. (I) WBC count per µl (left) and percentage of GFP (BCR-ABL1)$^+$ BP1$^+$ cells (right) of all cells in the peripheral blood of WT (black) or tPA-deficient (tPA KO) (gray) recipient mice with BCR-ABL1$^+$ B-ALL on day 25 after transplantation (P = 0.0023, two-tailed t-test, P = 0.0079, two-tailed Mann Whitney test, n=5, mean ± SD). (J) WBC count per µl (left) and percentage of GFP (BCR-ABL1)$^+$ BP1$^+$ cells (right) of all cells in the peripheral blood of WT secondary recipient mice of unsorted bone marrow cells from WT (black) or tPA KO (gray) mice with established BCR-ABL1$^+$ B-ALL on day 33 after transplantation (P = 0.030, two-tailed Mann Whitney test, n=9, mean ± SD). (K) Kaplan-Meier-style survival curve of WT secondary recipient mice of 3×10^6 unsorted bone marrow cells from WT (black) or tPA KO (gray) donor mice with established BCR-ABL1$^+$ B-ALL (P = 0.01, Log-rank test, n=9). (L) Quantification of fibronectin levels in IF images of bone sections from WT or ANXA2 KO recipient mice with BCR-ABL1$^+$ B-ALL on day 20 after transplant. Vehicle or tPA (10mg/kg) had been administered by intrafemoral (i.f.) injection 24h prior to sacrifice (P < 0.0001, P = 0.0132, two-way ANOVA, Sidak test, n=5, mean ± SD). Source data are provided as a Source Data file.

Supplemental Figure 13

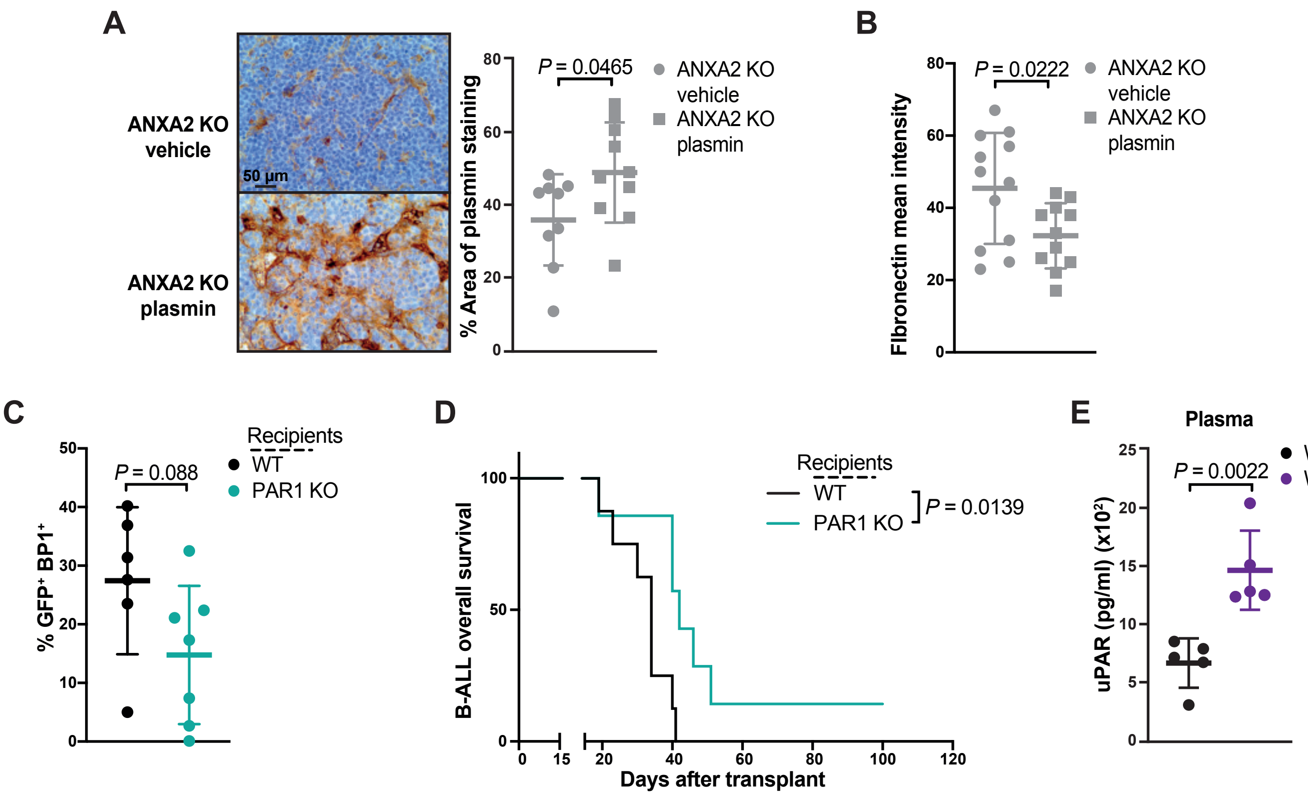

Supplemental Figure 13. (A) Immunohistochemistry (left) for plasmin (detected by immunoperoxidase using yellow-brown horseradish-peroxidase chromogen) and its quantification (right) in bone sections of ANXA2 KO recipient mice treated with vehicle (PBS) (circles) or plasmin (1 mg/kg) (squares) weekly starting from day 2 after transplantation with BCR-ABL1$^+$ B-ALL-initiating cells (P = 0.0465, two-tailed t-test, vehicle n=9, plasmin n=10, mean ± SD). The scale bar depicts 50 µm. (B) Quantification of fibronectin levels in IF images of bone sections of ANXA2 KO recipient mice treated with vehicle (PBS) (circles) or plasmin (1 mg/kg) (squares) weekly starting from day 2 after transplantation with BCR-ABL1$^+$ B-ALL-initiating cells (P = 0.0222,two-tailed t-test, n=3, mean ± SD). (C) Percentage of GFP (BCR-ABL1)$^+$ BP1$^+$ cells of all cells in the peripheral blood of WT (black) or protease-activated receptor 1 deficient (PAR1 KO) (light blue) recipient mice with BCR-ABL1$^+$ B-ALL on day 26 after transplantation (P = 0.088, two-tailed t-test, WT n=6, PAR1 KO n=7, mean ± SD). (D) Kaplan-Meier-style survival curve of WT (black) or PAR1 deficient (PAR1 KO) (light blue) recipient mice with BCR-ABL1$^+$ B-ALL (P = 0.0139, Log-rank test, WT n=8, PAR1 KO n=7). (E) Levels of urokinase-type plasminogen activator receptor (uPAR), measured by ELISA in the BM supernatant from healthy WT mice (black) or WT recipient mice with BCR-ABL1+ B-ALL (purple) (P = 0.0022, two-tailed t-test, n=5, mean ± SD). The mice were unirradiated. Source data are provided as a Source Data file.

Supplemental Figure 14

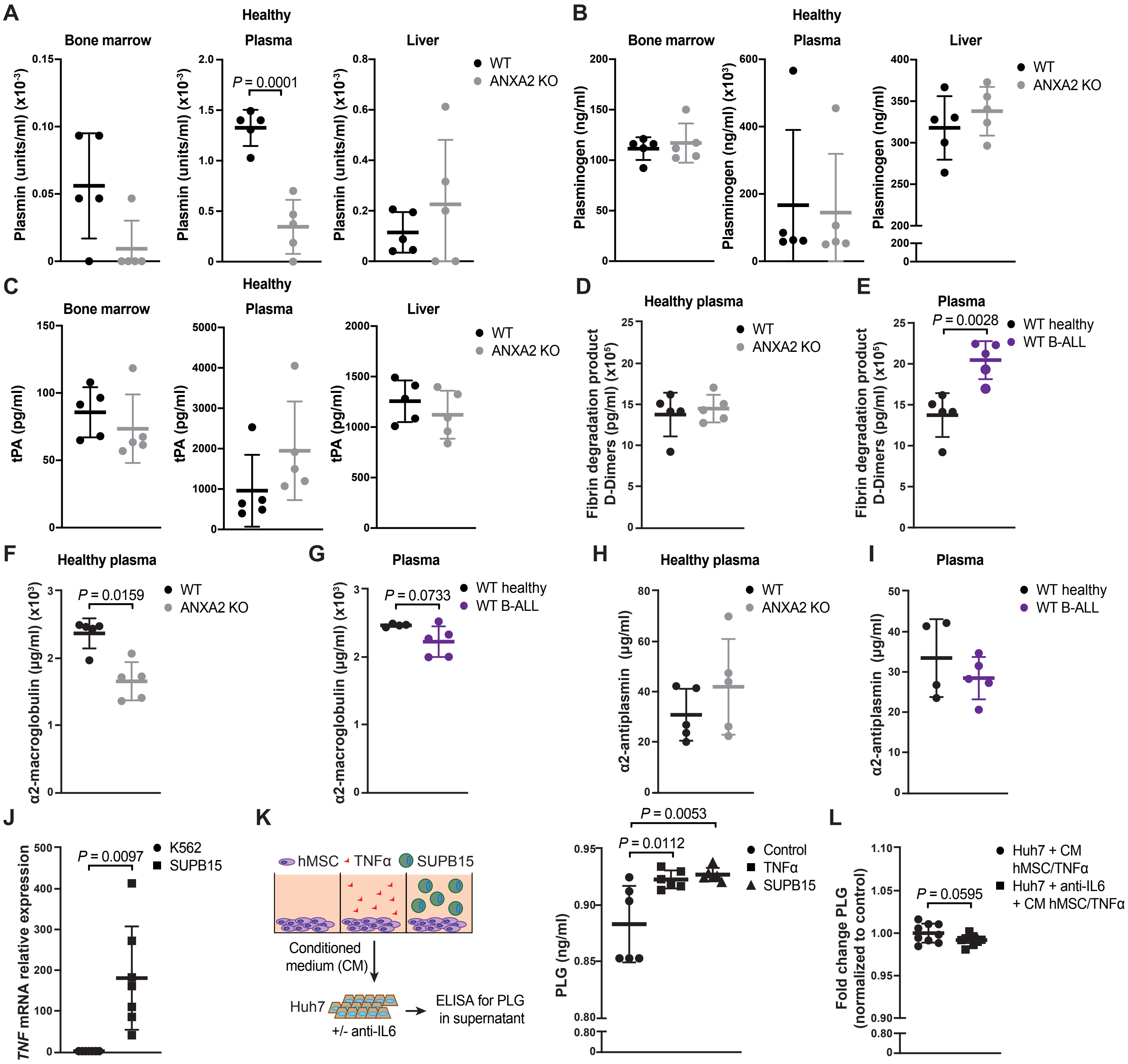

Supplemental Figure 14. (A) Levels of active plasmin, measured as the ability to cleave a colorogenic substrate 2h after addition of the substrate to the supernatants of BM, plasma or crushed liver from healthy WT (black) or ANXA2 KO (gray) mice (P = 0.0001, two-tailed t-test, n=5, mean ± SD). (B) Levels of plasminogen, measured by ELISA in the supernatants of BM, plasma or crushed liver from healthy WT (black) or ANXA2 KO (gray) mice (n=5, mean ± SD). We cannot exclude that the employed antibody also detects plasmin. (C) Levels of tissue plasminogen activator (tPA), measured by ELISA in the supernatants of BM, plasma or crushed liver from healthy WT (black) or ANXA2 KO (gray) mice (n=5, mean ± SD). (D) Levels of the fibrin degradation product D-Dimers, measured by ELISA in the plasma of healthy WT (black) or ANXA2 KO (gray) mice (n=5, mean ± SD). (E) Levels of the fibrin degradation product D-Dimers, measured by ELISA in the plasma of healthy WT mice (black) or WT recipient mice with BCR-ABL1$^+$ B-ALL (purple) (P = 0.0028, two-tailed t-test n=5, mean ± SD). (F) Levels of α2-macroglobulin, measured by ELISA in the supernatants of BM, plasma or crushed liver of healthy WT (black) or ANXA2 KO (gray) mice (P = 0.0159, two-tailed t-test, n=5, mean ± SD). (G) Levels of α2-macroglobulin, measured by ELISA in the plasma of healthy WT mice (black) or WT recipient mice with BCR-ABL1$^+$ B-ALL (purple) (P = 0.0733, two-tailed t-test, n=5, mean ± SD). (H) Levels of α2-antiplasmin, measured by ELISA in the supernatants of BM, plasma or crushed liver from healthy WT (black) or ANXA2 KO (gray) mice (n=5, mean ± SD). (I) Levels of α2-

antiplasmin, measured by ELISA in the plasma of healthy WT mice (black) or WT recipient mice with BCR-ABL1$^+$ B-ALL (purple) (n=5, mean ± SD). (J) Relative expression of TNF in the BCR-ABL1$^+$ CML human cell line, K562, or in the BCR-ABL1$^+$ B-ALL human cell line SUPB15, as measured by qRT-PCR (*P* = 0.0097, two-tailed *t*-test, n=7, mean ± SD). (K) Levels of plasminogen, measured by ELISA in the supernatants of Huh7 cells cultured with conditioned medium of primary human MSC exposed to vehicle, recombinant TNFα (15 ng/ml) or SUPB15 (*P* = 0.0053 and *P* = 0.0112, one-way ANOVA, n=6, mean ± SD). (L) Levels of plasminogen, measured by ELISA in the supernatants of Huh7 cells cultured with conditioned medium of primary human MSC exposed to TNFα, in presence or absence of anti-IL6. The fold change is shown normalized over Huh7 + CM hMSC/TNFα (control) (*P* = 0.0595, two-tailed *t*-test, n=5, mean ± SD). We cannot exclude that the employed antibody also detects plasmin. Source data are provided as a Source Data file.

Supplemental Figure 15

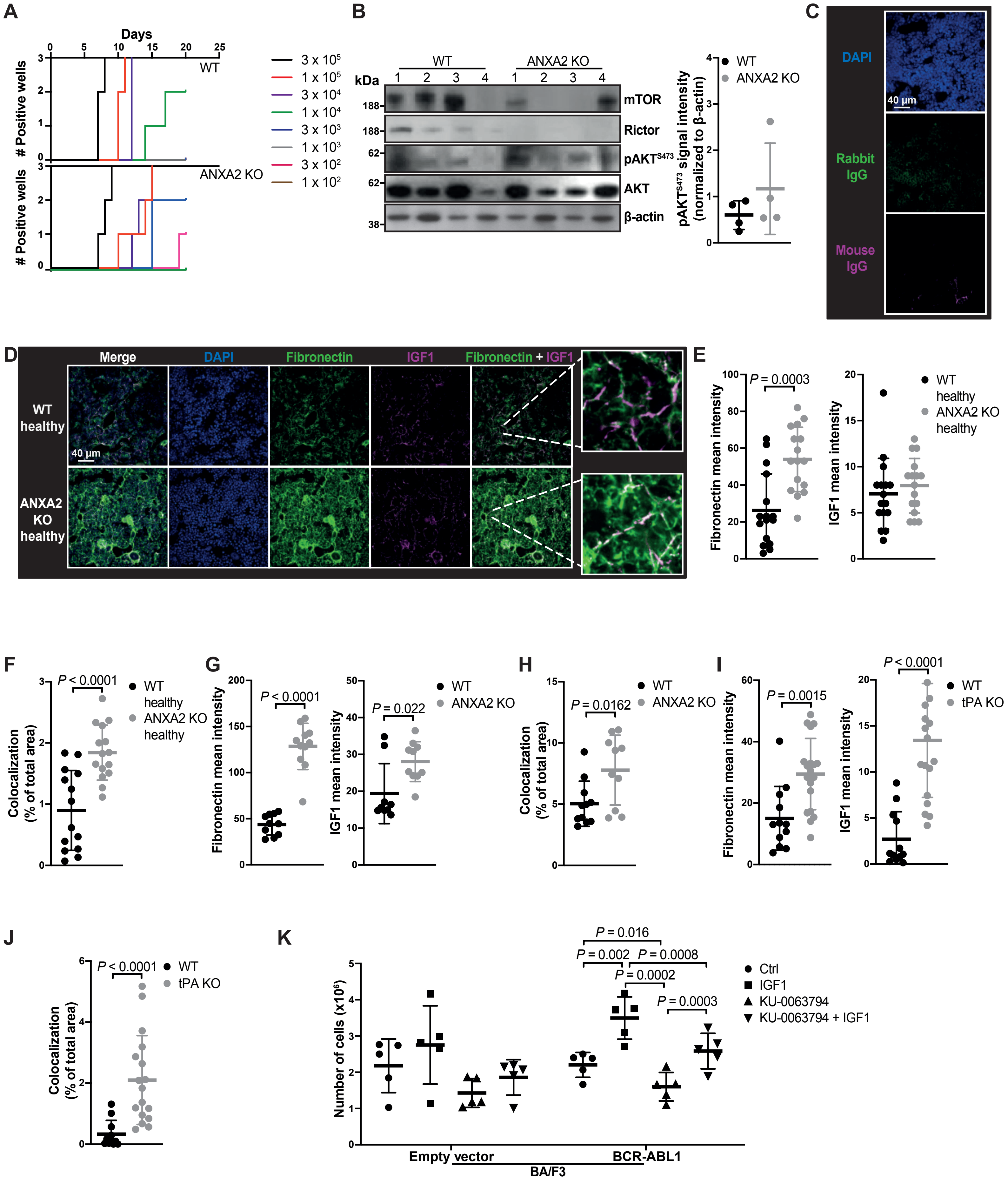

Supplemental Figure 15. (A) Serial dilutions of BCR-ABL1-transduced total bone marrow cells (indicated by the line color) were plated in triplicate on WT (top) or ANXA2 KO (bottom) MSC and cultured for 3 weeks. The number of replicates (3 in total per dilution) that reached confluence (≥$1x10^6$ non-adherent cells) is indicated on the y-axis. The time in days to reach confluence is shown on the x-axis. (B) Immunoblot analysis of unsorted spleen cells from WT or ANXA2 KO recipient mice with BCR-ABL1$^+$ B-ALL for the indicated proteins. Samples were collected on day 20 after transplantation, and each column represents a single mouse. The quantification on the right is for the band intensity of pAKT^{S473} normalized over the loading control β-actin (n=4, mean ± SD). (C) Immunofluorescence (IF) images of bone sections from WT mice stained with anti-rabbit (green) or -mouse (purple) IgG control antibodies and DAPI. The scale bar depicts 40 µm. (D) IF images of bone sections from normal WT or ANXA2 KO mice, stained for fibronectin (green) and IGF1 (purple). In the magnified images the white areas indicate the colocalization of fibronectin and IGF1 (n=4). The scale bar depicts 40 µm. (E) Quantification of fibronectin (left) or IGF1 (right) levels in IF images of bone sections from normal WT (black) or ANXA2 KO (gray) mice. Each dot represents an image (P = 0.0003, two-tailed Mann Whitney test, n=4, mean ± SD). (F) Quantification of the colocalization of fibronectin and IGF1 in bone sections from normal WT (black) or ANXA2 KO (gray) mice. Each dot represents an image (P < 0.0001, two-tailed *t*-test, n=4, mean ± SD). (G) Quantification of fibronectin (left) or IGF1 (right) levels in IF images of bone sections of WT (black) or ANXA2 KO (gray) mice with BCR-ABL1$^+$ B-ALL on day 20 after transplantation. Each dot represents an image (P < 0.0001, two-tailed *t*-test, P = 0.022, two-tailed Mann Whitney test, n=3 mice, mean ± SD). (H) Quantification of the colocalization of fibronectin and IGF1 in bone sections of WT (black) or ANXA2 KO (gray) mice with BCR-ABL1$^+$ B-ALL on day 20 after transplantation. Each dot represents an image (P = 0.0162, two-tailed *t*-test, n=3, mean ± SD). (I) Quantification of fibronectin (left) or IGF1 (right) levels in IF images of bone sections of WT (black) or tPA KO (gray) mice with BCR-ABL1$^+$ B-ALL on day 20 after transplantation. Each dot represents an image (P = 0.0015, *t*-test, P < 0.0001, two-tailed Mann Whitney test, n=3, mean ± SD). (J) Quantification of the colocalization of fibronectin and IGF1 in bone sections of WT (black) or tPA KO (gray) mice with BCR-ABL1$^+$ B-ALL on day 20 after transplantation. Each dot represents an image (P < 0.0001, two-tailed Mann Whitney test, n=3, mean ± SD). (K) Proliferation of empty vector$^+$ or BCR-ABL1$^+$ BA/F3 cells after 48h of culture in medium containing murine recombinant insulin-like growth factor (IGF1) (12 ng/ml) with or without the mTOR inhibitor KU-0063794 (10 nM) (P = 0.016, P = 0.002, P = 0.0008, P = 0.0002, P = 0.0003, two-way ANOVA, Tukey test, n=5, mean ± SD). Source data are provided as a Source Data file.

Supplemental Figure 16

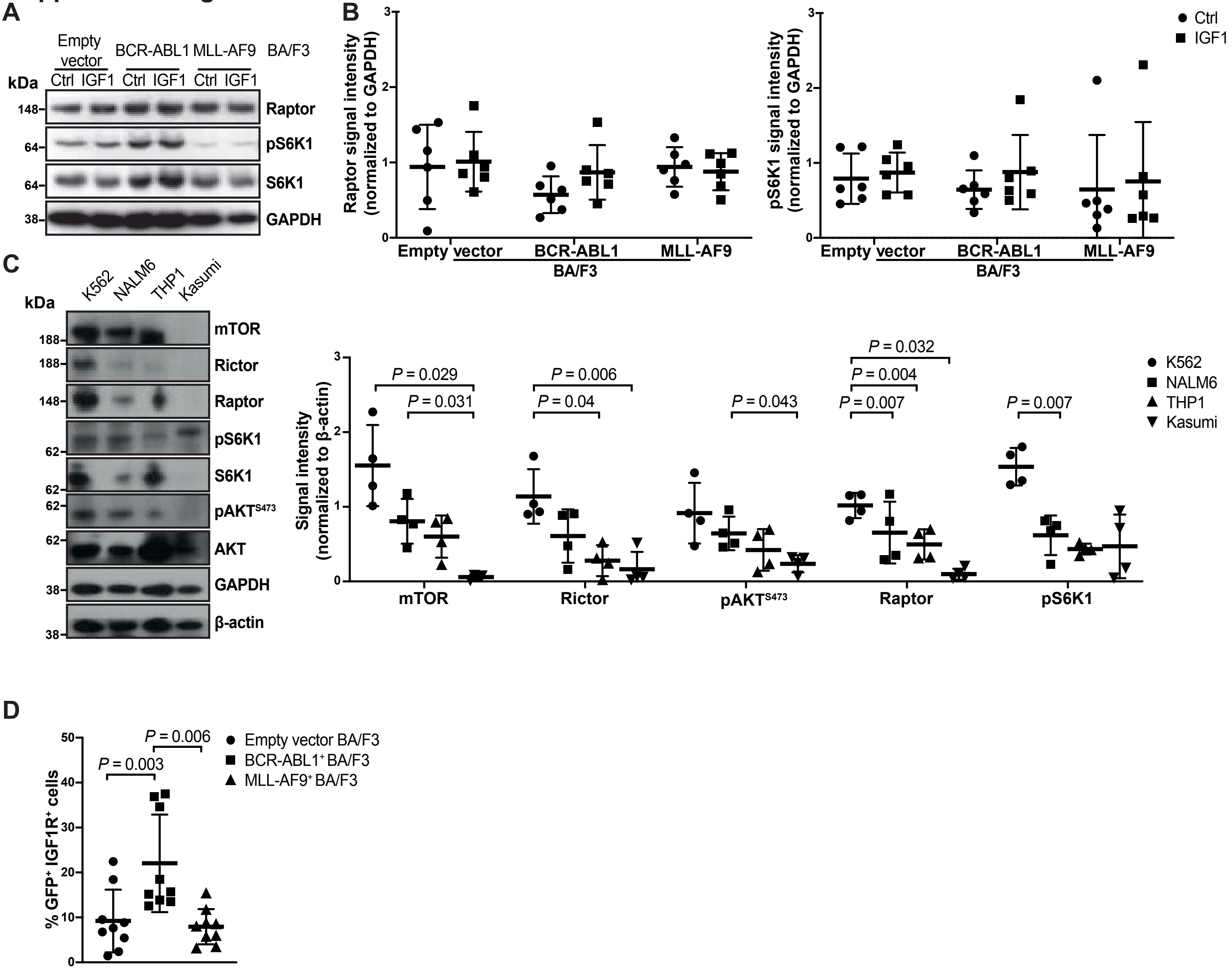

Supplemental Figure 16. (A) Representative immunoblot for the indicated proteins in lysates of empty vector$^+$, BCR-ABL1$^+$ or MLL-AF9$^+$ BA/F3 cells treated for 5 minutes with murine recombinant IGF1 (12 ng/ml) (n=6). (B) Quantification of the band intensities for Raptor and pS6K1 from (A), normalized by GAPDH (n=6, mean ± SD). (C) Representative immunoblot for the indicated proteins in lysates of K562, NALM6, THP1 and Kasumi cells at baseline (left) and quantification (right) of the band intensities for mTOR, Rictor, pAKT^{S473}, Raptor and pS6K1, normalized by β-actin (P = 0.029, P = 0.031, P = 0.006, P = 0.04, P = 0.043, P = 0.032, P = 0.004, P = 0.007, P = 0.007, two-way ANOVA, Dunnett´s test, n=4, mean ± SD). The samples are derived from the same experiment, but one gel was used for mTOR, Rictor, pAKT^{S473}, AKT and β-actin. Another gel was used for Raptor, pS6K1 and S6K1. (D) Percentage of GFP$^+$ IGF1R$^+$ cells of all empty vector$^+$, BCR-ABL1$^+$ or MLL-AF9$^+$ BA/F3 cells (P = 0.003, P = 0.006, Friedman test, Dunn test, n=9, mean ± SD). Source data are provided as a Source Data file.

Supplemental Figure 17

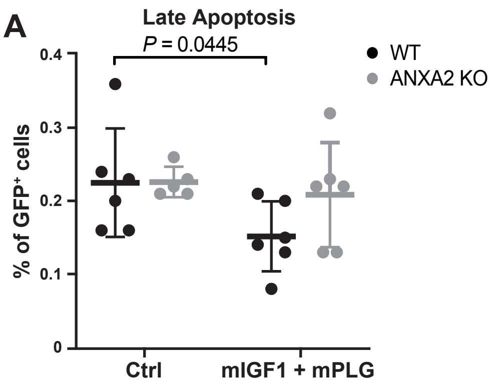

Supplemental Figure 17. (A) Percentage of late apoptotic cells of sorted primary GFP (BCR-ABL1)$^+$ cells from the BM of WT mice transduced with BCR-ABL1-expressing retrovirus after 48h of culture. Cells were plated on either WT (black) or ANXA2 KO (gray) MSC embedded in matrigel. IGF1 (mIGF1) and plasminogen (mPLG) were embedded in matrigel (mIGF1) (P = 0.0445, two-way ANOVA, Tukey test, n=6, mean ± SD). Source data are provided as a Source Data file.

Supplemental Figure 18

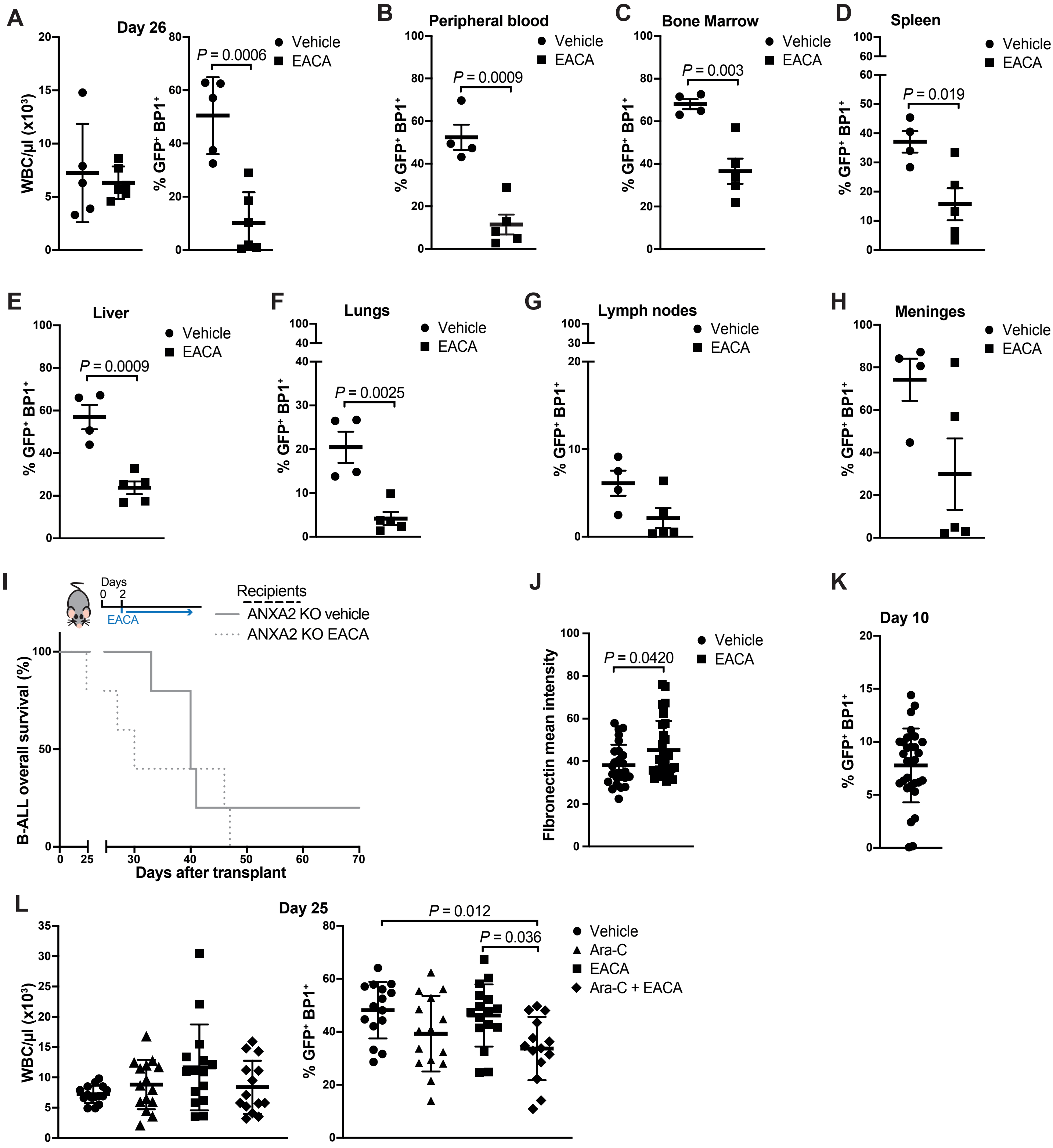

Supplemental Figure 18. (A) WBC count per μl (left) and percentage of GFP (BCR-ABL1)⁺ BP1⁺ cells (right) of all cells in the peripheral blood of WT recipient mice with BCR-ABL1⁺ B-ALL treated with vehicle (PBS) (circles) or EACA (1.2 mg/kg daily) (squares) starting from day 2 after transplant (P = 0.0006, two-tailed t-test, vehicle n=5, EACA n=6, mean ± SD). The blood was collected on day 26 after transplantation. (B-H) Percentage of GFP (BCR-ABL1)⁺ BP1⁺ cells of all cells in the peripheral blood (B), bone marrow (C), spleen (D), liver (E), lungs (F), lymph nodes (G) and meninges (H) of WT recipient mice with BCR-ABL1⁺ B-ALL treated with vehicle (PBS) (circles) or EACA (1.2 mg/kg daily) (squares) starting from day 2 after transplant (P = 0.0009, P = 0.003, P = 0.019, P = 0.0009, P = 0.0025, two-tailed t-test, n=4, mean ± SD). (I) Kaplan-Meier-style survival curve of ANXA2 KO recipient mice with BCR-ABL1⁺ B-ALL treated with vehicle (PBS) (solid line) or ε-aminocaproic acid (EACA) (1.2 mg/kg daily) (dotted line) starting from day 2 after transplant (Log-rank test, vehicle n=5, EACA n=6). (J) Quantification of fibronectin levels in immunofluorescence images of bone sections of WT recipient mice with BCR-ABL1⁺ B-ALL treated with vehicle (PBS) (circles) or EACA (1.2 mg/kg daily) (squares) starting from day 2 after transplant. The samples were obtained on day 20 after transplantation. The values of the WT vehicle samples are shared

with those from Supplemental Figure 15G. Each dot represents an image (P = 0.0420, two-tailed Mann Whitney Test, n=3, mean ± SD). (K) Percentage of GFP (BCR-ABL1)$^+$ BP1$^+$ cells of all cells in the peripheral blood of WT recipient mice with BCR-ABL1$^+$ B-ALL, collected on day 10 after transplantation prior to the start of treatment (n=30, mean ± SD). (L) WBC count per µl (left) and percentage of GFP (BCR-ABL1)$^+$ BP1$^+$ cells (right) of all cells in the peripheral blood of WT recipient mice with BCR-ABL1$^+$ B-ALL treated with vehicle (PBS), cytarabine (ara-C) (0.5 mg/kg on day 12 and 19), EACA (1.2 mg/kg daily) or a combination of ara-C (0.5 mg/kg on day 12 and 19) and EACA (1.2 mg/kg daily) as from day 10 after transplantation. The samples were collected on day 25 after transplantation, (P = 0.012, P = 0.036, one-way ANOVA, Tukey´s test, vehicle n=15, ara-C n= 15, EACA n=15, ara-C and EACA n=14, mean ± SD). Source data are provided as a Source Data file.

Supplemental Figure 19

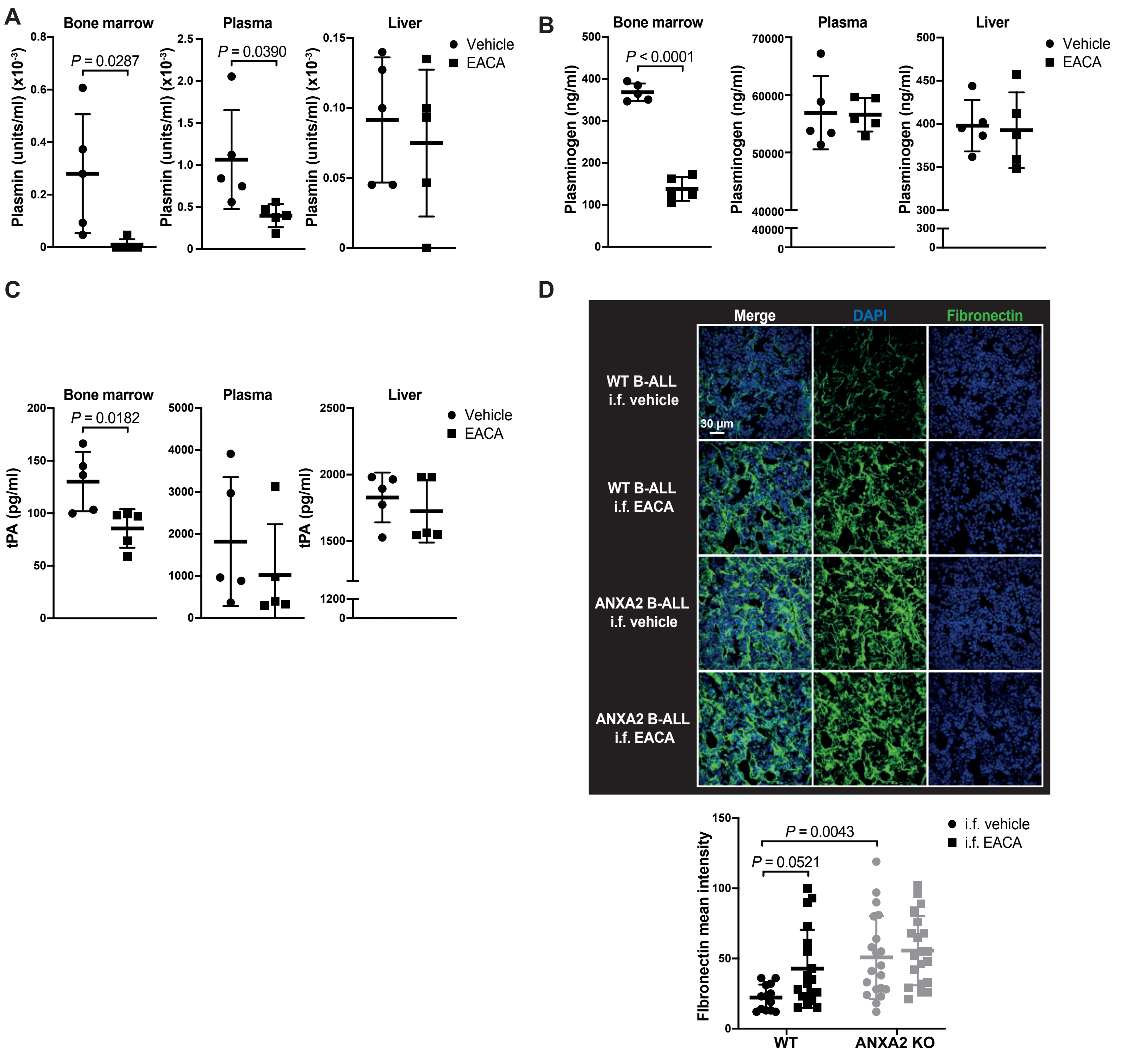

Supplemental Figure 19. (A) Levels of active plasmin, measured as the ability to cleave a colorogenic substrate 2h after addition of the substrate to supernatants of BM, plasma or crushed liver from WT recipient mice with BCR-ABL1⁺ B-ALL treated with vehicle (PBS) (circles) or EACA (1.2 mg/kg daily) (squares) starting from day 2 after transplant (*P* = 0.0159, two-tailed Mann Whitney test, n=5, mean ± SD). (B) Levels of plasminogen, measured by ELISA in supernatants of BM, plasma or crushed liver from WT recipient mice with BCR-ABL1⁺ B-ALL treated with vehicle (PBS) (circles) or EACA (1.2 mg/kg daily) (squares) starting from day 2 after transplant (*P* < 0.0001, two-tailed *t*-test, n=5, mean ± SD). (C) Levels of tissue plasminogen activator (tPA), measured by ELISA in the supernatants of BM, plasma or crushed liver from WT recipient mice with BCR-ABL1⁺ B-ALL treated with vehicle (PBS) (circles) or EACA (1.2 mg/kg daily) (squares) starting from day 2 after transplant (*P* = 0.0182, two-tailed *t*-test, n=5, mean ± SD). (D) Immunofluorescence images (top) and quantification (bottom) of bone sections, stained for fibronectin (green) and DAPI (blue), from WT or ANXA2 KO recipient mice with BCR-ABL1⁺ B-ALL on day 16 after transplant. Vehicle or EACA (1.2 mg/kg) had been administered by intrafemoral (i.f.) injection 24h prior to sacrifice (n=5). The scale bar represents 30 µm (*P* = 0.0521, *P* = 0.0043, two-way ANOVA, Sidak test, n=5, mean ± SD). Source data are provided as a Source Data file.

Supplemental Figure 20

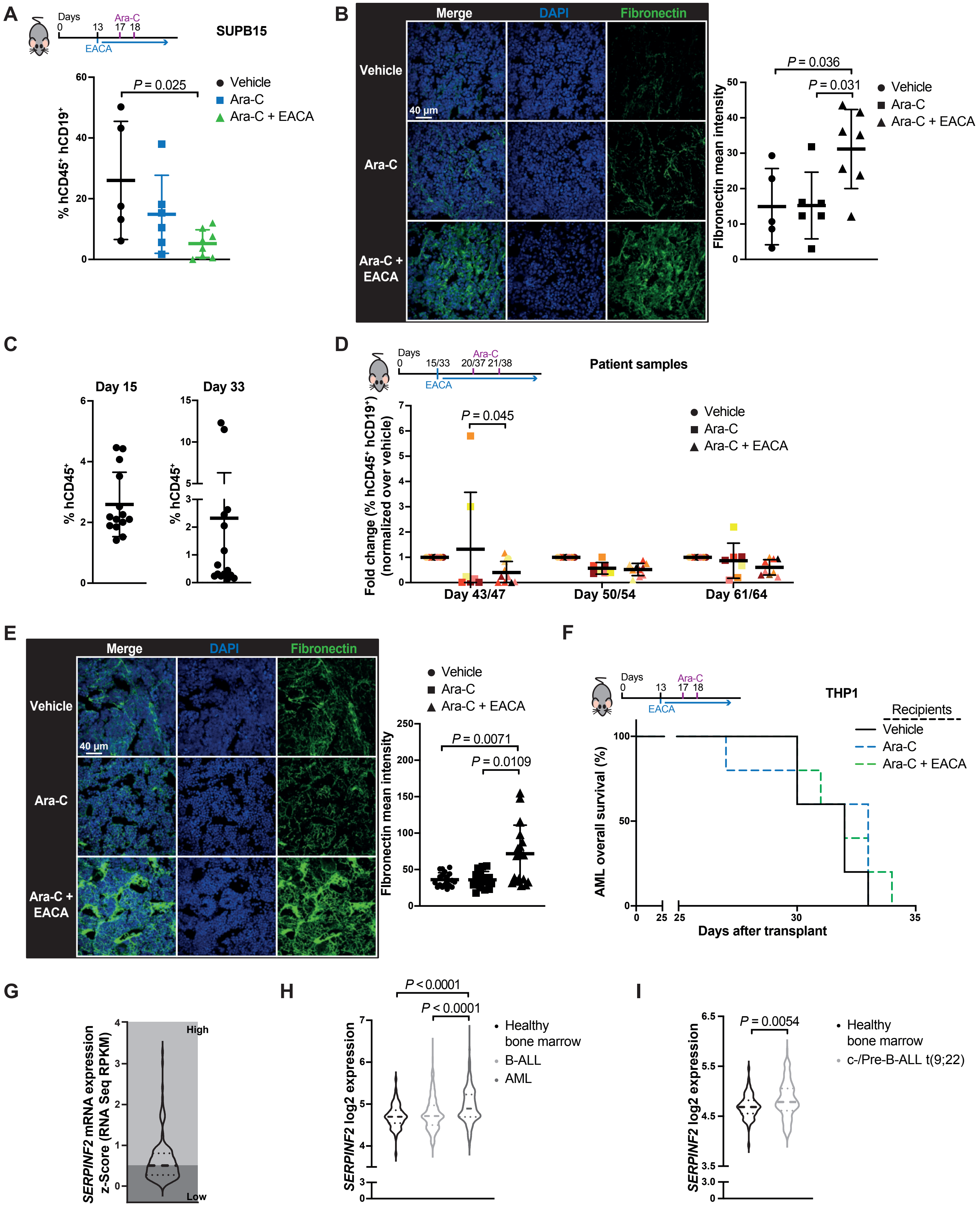

Supplemental Figure 20. (A) Percentage of human $CD45^+$ $CD19^+$ leukocytes of all cells in the peripheral blood of NOD SCID interleukin-2 receptor γ knockout (NSG) mice transplanted with $1x10^6$ SUPB15 cells and treated with vehicle, ara-C (75 mg/kg, on day 17 and 18) and a combination of ara-C (75 mg/kg, on day 17 and 18) and EACA (1.2 mg/kg, daily from day 13) (P = 0.025, one-way ANOVA, Tukey test, n=7, mean ± SD). (B) Representative immunofluorescence images (left) and their quantification (right) of bone sections stained for fibronectin (green) and DAPI (blue). The bone sections are from NSG mice transplanted with 1 x 10^6 SUPB15 cells and treated with vehicle, ara-C (75 mg/kg, on day 17 and 18) and a combination of ara-C (75 mg/kg, on day 17 and 18) and EACA (1.2 mg/kg, daily from day 13) (P = 0.036, P = 0.031, one-way ANOVA, n=7, mean ± SD). (C) Percentage of $hCD45^+$ cells of all cells in the peripheral blood of NSG recipient mice transplanted with $1.5\text{-}2x10^6$ cells from 6 patients with B-ALL (peripheral blood or bone marrow), collected on day 15 or 33 after transplantation (n=14-15, mean ± SD). (D) Fold change (normalized to the matching vehicle) of the percentage of human $CD45^+$ $CD19^+$ leukocytes of all cells in the peripheral blood of NSG mice transplanted with $1.5\text{-}2x10^6$ cells from 6 patients with B-ALL. 1 patient sample is BCR-$ABL1^+$ and 5 are BCR-$ABL1^-$. Further genotypic information was not available. Each sample was transplanted into at least 3 mice, whereby one mouse was treated with vehicle (circles), one with ara-C (75 mg/kg) (squares) and one with a combination of ara-C (75 mg/kg) and EACA (1.2 mg/kg daily) (triangle). EACA treatment started on day 15 or day 33 after transplantation, i.e. once $CD45^+$ $CD19^+$ cells were detected in the peripheral blood of transplanted mice (P = 0.045, two-way ANOVA, Tukey test, n=6, mean ± SD). (E) Representative IF images (left) and their quantification (right) of bone sections from NSG mice, stained for fibronectin (green) and DAPI (blue). The NSG mice had been transplanted with $1.5x10^6$ cells from 4 patients with B-ALL (peripheral blood or bone marrow) and treated with vehicle (PBS) (circles), ara-C (75 mg/kg) (squares) or a combination of ara-C (75 mg/kg) and EACA (1.2 mg/kg daily) (triangles) as in D (P = 0.0071and P = 0.0109, Kruskal Wallis test, Dunn test, vehicle n=5, ara-C n=4, ara-C and EACA n=6, mean ± SD). (F) Kaplan-Meier-style survival curve of NOD SCID interleukin-2 receptor γ knockout (NSG) mice transplanted with 1 x 10^6 THP1 cells (MLL-$AF9^+$), treated with vehicle (PBS; black), ara-C (50 mg/kg; blue) or with a combination of ara-C (50 mg/kg) and EACA (1.2 mg/kg daily) (green). EACA treatment was started on day 13 after transplantation, while ara-C was administered on days 17 and 18 after transplantation (n=5). (G) Expression levels of *SERPINF2* (α2-antiplasmin) in human patients with B-ALL (n=71). The average expression level was chosen as the cutoff for high versus low expression. The data were generated using the publicly available dataset TARGET, accessed through the cBioPortal portal. RPKM = reads per kilobase of transcript per million reads mapped. (H) Log2 expression of *SERPINF2* in BM cells of healthy individuals (n=73), patients with B-ALL (n=576) or AML (n=542), taken from the BloodSpot portal (MILE study) (P < 0.0001, Kruskal-Wellis test, mean ± SD). (I) Log2 expression of *SERPINF2* in BM cells of healthy individuals (n=73) or patients with c-/pre-B-ALL (n=122) expressing the BCR-ABL1 oncoprotein (t(9;22)) taken from the BloodSpot portal (MILE study) (P = 0.0054,two-tailed t-test). Source data are provided as a Source Data file.

Supplemental Figure 21

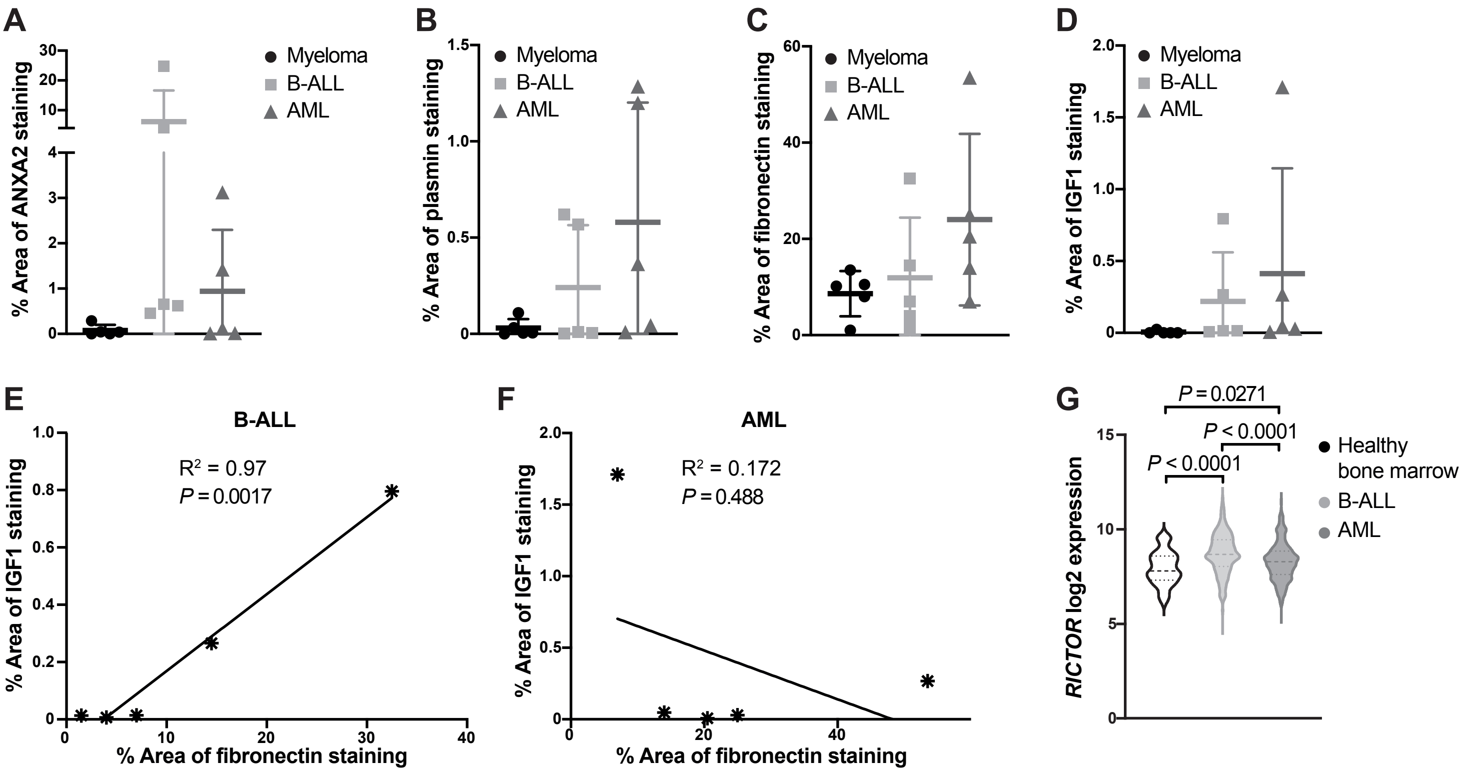

Supplemental Figure 21. (A-D) Quantification of the immunohistochemistry (IHC) images of bone sections from patients with multiple myeloma (circles), B-ALL (squares) or AML (triangles) stained for ANXA2 (A), plasmin (B), fibronectin (C) or IGF1 (D) (detected by immunoperoxidase using yellow-brown horseradish-peroxidase chromogen) (n=5, mean ± SD). (E-F) Correlation between fibronectin and IGF1 levels from the IHC staining in figure 8D in each patient with B-ALL (E) or AML (F). (G) Log2 expression of *RICTOR* in the BM cells of healthy individuals (n=73; circles), patients with B-ALL (n=576; squares) or AML (n=542; triangles), taken from the BloodSpot portal (MILE study) ($P < 0.0001$, $P = 0.0271$ and $P < 0.0001$, one-way ANOVA Tukey test, mean ± SD). Source data are provided as a Source Data file.

Supplemental Figure 22

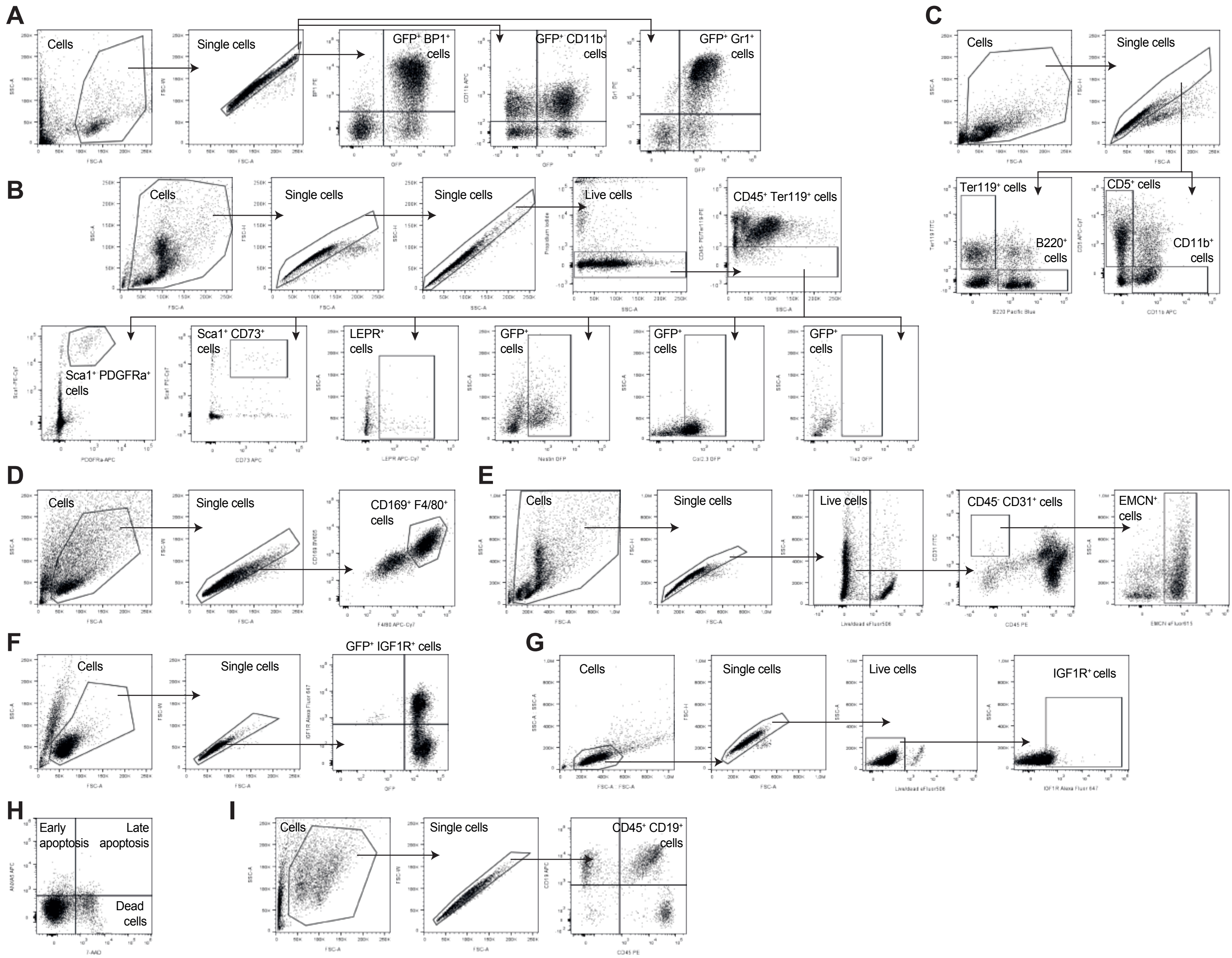

Flow cytometry gating strategies. (A) Gating strategy to identify GFP$^+$ BP1$^+$ leukemia cells in mice with B-ALL (Figures 1A and 1D), GFP$^+$ CD11b$^+$ leukemia cells in mice with CML (Supplemental Figure 5A) and GFP$^+$ Gr1$^+$ leukemia cells in mice with AML (Supplemental Figure 5F). (B) Gating strategy for sorting Sca1$^+$ PDGFRa$^+$ MSC, Sca1$^+$ CD73$^+$ MSC, LEPR$^+$ MSC, Nestin$^+$ MSC, Col2.3$^+$ osteoblastic cells and Tie$^+$ endothelial cells. (C) Gating strategy to identify Ter119$^+$ erythrocytes, B220$^+$ B cells, CD11b$^+$ myeloid cells and CD5$^+$ T cells (Supplemental Figures 2D-F). (D) Gating strategy to identify CD169$^+$ F4/80$^+$ macrophages (Supplemental Figure 11D). (E) Gating strategy to identify CD45$^-$ CD31$^+$ EMCN$^+$ endothelial cells (Supplemental Figure 11H). (F-G) Gating strategy to identify mouse GFP$^+$ IGF1R$^+$ cells (F) (Figure 5D) and human IGF1R$^+$ cells (G) (Figure 5E). (H) Gating strategy to identify ANXA5$^+$ 7-AAD$^+$ late apoptotic cells (Supplemental Figure 17). (I) Gating strategy to identify CD45$^+$ CD19$^+$ B-ALL cells in the peripheral blood of NSG mice transplanted with human B-ALL cells (Supplemental Figures 20A and 20D).

Antibody	Conjugation	Company	Catalogue number
Anti-mouse CD45R/B220	Biotin	BD Pharmingen	553086
Anti-mouse Ter119	Biotin	BD Pharmingen	553672
Anti-mouse CD5	Biotin	BD Pharmingen	553019
Anti-mouse Ter119	PE	BD Pharmingen	553673
Anti-mouse CD34	PE	BD Pharmingen	551387
Anti-mouse CD45	PE	BD Pharmingen	553081
Anti-human CD45	PE	BD Pharmingen	555483
Anti-mouse CD31	PE	BD Pharmingen	553373
Anti-mouse CD105	PECF594	BD Pharmingen	562762
Anti-mouse CD11b	APC	BD Pharmingen	553312
Anti-mouse c-Kit	APC	BD Pharmingen	553356
Anti-mouse Gr1	APC-Cy7	BD Pharmingen	557661
Anti-mouse CD16/32	V450	BD Pharmingen	560539
Anti-mouse CD90.1(Thy1.1)	BV711	BD Pharmingen	553772
Anti-mouse CD127(IL7Rα)	BV711	BioLegend	135035
Anti-human CD19	APC	BioLegend	302212
Annexin5	APC	BioLegend	640941
Streptavidin	APC-Cy7	BioLegend	405208
Anti-mouse CD48	PB	BioLegend	103418
Anti-mouse CD44	PB	BioLegend	103020
Anti-mouse CD29	FITC	BioLegend	102206
Anti-mouse Ly-51(BP1)	PE	BioLegend	108308
Anti-mouse Flk2(CD135)	PE	BioLegend	135305
Anti-mouse CD150	PE	BioLegend	115904
Anti-mouse Sca-1	PE-Cy7	BioLegend	108114
Anti-mouse CD51	biotin	BioLegend	13-0512-22
Anti-mouse PDGFRα	APC	ThermoFisher	17-1401-81
Anti-human F4/80	Biotin	ThermoFisher	MF48015
Anti-mouse/human ANXA2	Rabbit	Cell Signaling	8235
Anti-mouse/human mTOR	Rabbit	Cell Signaling	2983
Anti-mouse/human Rictor	Rabbit	Cell Signaling	2114
Anti-mouse/human Raptor	Rabbit	Cell Signaling	2280
Anti-mouse/human phospho-Akt Ser473	Rabbit	Cell Signaling	9271
Anti-mouse/human phospho-Akt Thr308	Rabbit	Cell Signaling	9275
Anti-mouse/human Akt	Rabbit	Cell Signaling	9272
Anti-mouse/human phospho-CrkI Thr207	Rabbit	Cell Signaling	3181
Anti-mouse/human phospho -p70 S6 Kinase Thr389	Rabbit	Cell Signaling	9234
Anti-mouse/human p70 S6 Kinase	Rabbit	Cell Signaling	9202
Anti-mouse/human IGF1 Receptor β	Rabbit	Cell Signaling	9750
Anti-mouse/human CREB	Rabbit	Cell Signaling	9197

Antibody	Conjugation	Company	Catalogue number
IgG	Rabbit	Cell Signaling	2729
Anti-rabbit IgG	HRP	Cell Signaling	7074
Anti-mouse IgG	HRP	Cell Signaling	7076
Anti-mouse IGF1	Mouse	Abcam	ab212477
Anti-human IGF1	Mouse	Abcam	ab176523
Anti-mouse Crkl	Rabbit	Abcam	ab151791
Anti-mouse vimentin	Rabbit	Abcam	ab92547
Anti-mouse/human fibronectin	Rabbit	Abcam	ab2413
Anti-mouse laminin	Rabbit	Abcam	ab11575
Anti-human plasmin	Biotin	Abcam	ab48350
Anti-mouse CD31	FITC	Invitrogen	11-0311-82
Anti-mouse CD4	eFluor450	Invitrogen	14-5851-82
Anti-mouse CD8	eFluor450	Invitrogen	48-0081-82
Anti-mouse Ter119	eFluor450	Invitrogen	48-5921-82
Anti-mouse CD11b	eFluor450	Invitrogen	48-0112-82
Anti-mouse Gr1	eFluor450	Invitrogen	48-5931-82
Anti-mouse CD31	eFluor450	Invitrogen	48-0311-82
Anti-mouse ECMN	Rat	Invitrogen	14-5851-82
Anti-rat IgG	eFluor615	Invitrogen	42-4817-80
Streptavidin	APC	Invitrogen	17-4917-82
Anti-mouse S100A10	Rabbit	Invitrogen	MA5-24080
Anti-mouse αSMA	Mouse	Invitrogen	14-9760-82
Anti-mouse/human vinculin	Mouse	Invitrogen	MA5-11690
Anti-rabbit	Alexa Fluor 488	Invitrogen	A-21206
Anti-rabbit	Alexa Fluor 647	Invitrogen	A-32733
Anti-mouse	Alexa Fluor 647	Invitrogen	A-32728
Anti-mouse GAPDH	Rabbit	Santa Cruz	sc-32233
Anti-mouse/human ANXA2	Mouse	Santa Cruz	sc-28385
Anti-mouse/human actin	Mouse	Sigma-Aldrich	A4700-100UL

Supplemental Table 1. Antibodies used in this study.

Gene	Forward primer	Reverse primer
Mouse *Anxa2*	5’-CAGCCTGGAGGGTGATCATT-3’	5’-CACATTGCTGCGGTTTGTCA-3’
Mouse *Gapdh*	5’-AGGTCGGTGTGAACGGATTTG-3’	5’-GGGGTCGTTGATGGCAACA-3’
Human *PLG*	5’-TGCAGCAAAATGTGAGGAGGA-3’	5’-TTGCACTCTGAGAGATACACTTTC-3’
Human *TNF*	5’-TGCACTTTGGAGTGATCGGC-3’	5’-CTCAGCTTGAGGGTTTGCTAC-3’
Human *GAPDH*	5’-GATCATCAGCAATGCCTCCT-3’	5’-GGACTGTGGTCATGAGTCCT-3’

Supplemental Table 2. List of primers used for quantitative RT-PCR in this study.

Primer name	Primer sequence (3´- 5´)
mAnxa2 s	CACCGAAACCGCAGCAATGTGCAG
mAnxa2 as	AAACCTGCACATTGCTGCGGTTTC
NTC s	CACCGTTCCGGGCTAACAAGTCCT
NTC as	AAACAGGACTTGTTAGCCCGGAAC
LKO.1	GACTATCATATGCTTACCGT

Supplemental Table 3. List of primers used in the cloning of sgRNA constructs. s = sense; as = antisense.

Gene	Forward primer	Reverse primer
Human *PLG* set1	5'-TTTGAACCCTGCTGAGCCAG-3'	5'-AGAAAGTGGGTCCCAATCCC-3'
Human *PLG* set2	5'-GGCATGGGTCTCTGAGAGAATC-3'	5'-CCAGCAGTGCCCAGAAAGT-3'

Supplemental Table 4. List of primers used for chromatin immunoprecipitation (ChIP) in this study.
